# Preclinical and clinical evidence for the treatment of non-alcoholic fatty liver disease with soybean: A systematic review and meta-analysis

**DOI:** 10.3389/fphar.2023.1088614

**Published:** 2023-01-25

**Authors:** Yubing Li, Xinyu Deng, Xiaochuan Guo, Fangling Zhang, Hefei Wu, Xuhua Qin, Xiao Ma

**Affiliations:** ^1^ State Key Laboratory of Southwestern Chinese Medicine Resources, School of Pharmacy, Chengdu University of Traditional Chinese Medicine, Chengdu, China; ^2^ Hospital of Chengdu University of Traditional Chinese Medicine, School of Clinical Medicine, Chengdu University of Traditional Chinese Medicine, Chengdu, China

**Keywords:** soybean, NAFLD, meta-analysis, clinical, preclinical

## Abstract

Non-alcoholic fatty liver disease (NAFLD), a prevalent public health issue, involves the accumulation of triglycerides in hepatocytes, which is generally considered to be an early lesion of liver fibrosis and cirrhosis. Thus, the development of treatments for NAFLD is urgently needed. This study explored the preclinical and clinical evidence of soybeans to alleviate NAFLD. Studies indexed in three relevant databases—Web of Science, PubMed, and Embase—between January 2002 and August 2022 were retrieved. A total of 13 preclinical studies and five RCTs that included 212 animals and 260 patients were included in the present analysis. The preclinical analysis showed that liver function indices (AST, SMD = −1.41, *p* < 0.0001 and ALT, SMD = −1.47, *p* < 0.0001) were significantly improved in the soybean group compared to the model group, and fatty liver indicators (TG, SMD = −0.78, *p* < 0.0001; TC, SMD = −1.38, *p* < 0.0001) and that oxidative stress indices (MDA, SMD = −1.09, *p* < 0.0001; SOD, SMD = 1.74, *p* = 0.022) were improved in the soybean group. However, the five RCTs were not entirely consistent with the preclinical results; however, the results confirmed the protective effect on the liver. The results of the clinical RCTs showed that soybean significantly affected liver function, fatty liver, and oxidative stress indicators (ALT, SMD = −0.42, *p* = 0.006; TG, SMD = −0.31, *p* = 0.039; MDA, SMD = −0.76, *p* = 0.007). The current meta-analysis combined preclinical and clinical studies and verified that soybean could protect the liver in NAFLD by regulating lipid metabolism and oxidative stress factors *via* the Akt/AMPK/PPARα signaling pathway. Soybean might be a promising therapeutic agent for treating non-alcoholic fatty liver disease.

**Systematic Review Registration:** (https://www.crd.york.ac.uk/prospero/#myprospero), identifier (CRD42022335822).

## Highlights

1. This study systematically evaluated soybean as a treatment for NAFLD based on meta-analyses of preclinical and clinical data.
2. The hepatoprotective effect of activating AMPK through P13K/AKT to improve insulin resistance, lipid metabolism, and the oxidative stress signaling pathway was the key process by which soybean alleviated NAFLD.
3. Soybean showed consistent therapeutic effects on NAFLD in clinical studies and preclinical trials.

## 1 Introduction

Non-alcoholic fatty liver disease (NAFLD) is a prevalent public health issue, especially in developed countries (Angulo, 2002). In recent years, NAFLD has become the most common chronic liver disease worldwide, seriously affecting human health, and has been recognized as a common cause of cirrhosis, with an increasing global prevalence of approximately 25% in the adult population (Powell et al., 2021). The increasing prevalence of chronic liver disease is associated with NAFLD and is proportional to the increase in obesity worldwide (Sherif et al., 2016). Generally, 10–20 years after confirming fatty liver, approximately 10% of patients with non-alcoholic fatty liver will develop cirrhosis (Ekstedt et al., 2015). Fatty liver is generally considered an early lesion of liver fibrosis and cirrhosis. Non-alcoholic fatty liver gradually develops into liver fibrosis and cirrhosis, with consequent large burdens on society. The development of treatments for NAFLD is urgently needed. Thus, the prevention of non-alcoholic fatty liver is critical for reducing liver fibrosis and cirrhosis in patients. The main causes of non-alcoholic fatty liver disease are the excessive accumulation of triglycerides and fatty infiltration of the liver (Mathiesen et al., 1999). PPAR-α activation can reduce fat synthesis and promote fat metabolism by regulating fatty acid transport and promoting FFA entry into mitochondria for β-oxidation to regulate lipid absorption in the liver. Therefore, PPAR-α agonists reduce triglyceride levels in the blood and increase the catabolism of triglyceride-rich lipoproteins. Although several PPAR-α agonists have been developed, only a few have been successfully applied in the clinical setting. Overall, there remains a lack of specific drugs for the treatment of NAFLD (Pan et al., 2014; Lin et al., 2016). At present, NAFLD is typically treated with pioglitazone and glutathione. However, compared to conventional drugs, soybean is considered a medicine and homologous food substance that can be easily obtained in daily life and has fewer adverse effects than conventional drugs. Various components in soybean can reduce blood lipid, total cholesterol, low-density lipoprotein, and triglyceride levels through different pathways.

“Diseases enter by the mouth” is a Chinese idiom, and many studies have shown that poor eating habits are an important cause of non-alcoholic fatty liver disease (Zhu et al., 2022). In contrast, clinical results have shown that good eating habits are an effective way to improve NAFLD. For instance, a Mediterranean diet, which is characterized by large amounts of fiber, polyunsaturated fat, and antioxidants, represents a healthy dietary pattern. The “Seven Countries Study” by Keys and White showed that a Mediterranean diet could ameliorate steatosis and fatty liver disease (Abenavoli et al., 2018). Soybeans are the main component of the Mediterranean diet and are considered an effective treatment for non-alcoholic fatty liver disease. The value of soybean as a treatment for non-alcoholic fatty liver disease has not been examined; however, in recent years, interest has been increasing regarding healthy diets to prevent and treat chronic diseases. Naturally, soybean can be used as a treatment for NAFLD. In recent years, a study indicated that soybean and soybean products may serve as potential treatments to cure NAFLD (Khoury et al., 2015). Soybean has a long history of use as food in China and throughout Asia and is popular with consumers. Soybeans can provide healthy nutrients and are generally recognized by the public; moreover, they have also been approved by the FDA to reduce the risk of complex diseases such as chronic heart disease (Huang et al., 2016). Previous studies demonstrated that the main active ingredients of soybean were various isoflavones (Chatterjee et al., 2018). Phytochemicals belonging to the genus flavonoids effectively reduced fatty liver disease induced by a high-fat diet in rats (Xu et al., 2019).

However, due to the limited research on soybean, particularly regarding the use of soybean for the treatment of NAFLD, there is no clear evidence for its use. Therefore, we conducted a meta-analysis of preclinical and clinical studies to provide further ideas for the use of soybean as a treatment for NAFLD. Furthermore, the mechanism by which soybean products treat non-alcoholic fatty liver disease is unclear and requires further research.

## 2 Methods

Based on the PRISMA 2020 guidelines, we searched the Web of Science, PubMed, and Embase databases for studies on the use of soybean for the treatment of NAFLD. The retrieval time was set from January 2002 to August 2022. The search term was the treatment combined with the disease. The treatment search terms were “soybean,” “soy bean,” and “soybean,” while the disease search terms were “non-alcoholic fatty liver disease,” “NAFLD,” and “NASH” (Supplementary Table S1). This study was registered in the International Prospective Register of Systematic Reviews. The PROSPERO register ID is CRD42022335822 (https://www.crd.york.ac.uk/prospero/#myprospero).

### 2.1 Inclusion criteria

The inclusion criteria for preclinical studies were 1) experimental animal models only for NAFLD/NASH, 2) only soybean and soybean products included in the treatment group, with no other drugs used in combination; 3) outcome indicators of TC, TG, ALT, AST, HDL-C, LDL-C, MDA, SOD, FFA, TNF-α, and insulin; and 4) histomorphology to determine the degeneration of hepatocyte adipose tissue.

The inclusion criteria for clinical studies were 1) participants were patients with NAFLD; 2) interventions of soybean and soybean products, with no other drugs were used in combination; 3) control group administered active therapy, placebo, or no therapy; 4) outcome indicators of TC, TG, ALT, AST, HDL-C, LDL-C, MDA, BMI, body weight, and insulin; and 5) randomized clinical trials.

### 2.2 Exclusion criteria

Preclinical studies meeting the following criteria were excluded: 1) experimental group not administered soybean or soybean products; 2) experimental group administered drugs other than soybean or soybean products; 3) other chronic liver diseases or other diseases that could cause fatty liver disease in animals; 4) animal liver fat degeneration caused by alcohol; 5) missing data on primary and secondary outcomes; 6) unpublished data or duplicated literature; 7) reviews and conference reports; 8) non-English language.

RCTs meeting the following criteria were excluded: 1) experimental group not administered soybean or soybean products; 2) experimental group administered drugs other than soybean or the control group did not receive a placebo or active treatment; 3) other chronic liver diseases or other diseases that could cause fatty liver disease in patients; 4) missing data on primary and secondary outcomes; and 5) non-English language.

### 2.3 Data extraction

We extracted the following information from the included literature according to the Preferred Reporting Items for Systematic Reviews and Meta-Analyses (PRISMA) standard: 1) the first author and the year of publication; 2) the method of non-alcoholic fatty liver disease induction in animals; 3) animal species and numbers of animals in the experimental and model groups; 4) intervention methods in the experimental groups and the model groups; and 5) primary and secondary outcome measures in the included literature.

For the five RCTs, we extracted the following information: 1) the first author and the year of publication; 2) sex and age of patients with non-alcoholic fatty liver disease; 3) intervention methods in the control and experimental groups; 4) sample size and treatment duration; and 5) outcome indicators. For studies requiring data extraction from images, we used universal Desktop Ruler software to extract the corresponding outcome indicators.

### 2.4 Quality assessment

We used the SYstematic Review Center for Laboratory Animal Experimentation (SYRCLE) animal laboratory bias risk assessment tool in Review Manager 5.3 software to evaluate the quality of the preclinical literature on soybean treatment of NAFLD and the methodological quality. The SYRCLE tool comprises 10 items and the final evaluation results are “Yes,” “No,” and “uncertain”. “Yes” indicates a low risk of bias, “No” indicates a high risk of bias, and “uncertain” indicates an uncertain risk of bias. The quality evaluation for preclinical studies included the following 10: 1) the allocation sequence was generated or applied adequately; 2) the baselines of each group were the same; 3) the allocation concealment was sufficient; 4) the animals were randomly placed during the experiment; 5) the researchers were blinded; 6) the animals were selected at random; 7) the evaluator was blinded; 8) incomplete data were processed completely; 9) no selective results were reported for these studies; and (10) no other issues in the study that might lead to a high risk of bias.

For RCTs, the evaluation checklist of the Cochrane Library was used to evaluate the study quality. This checklist contained the following seven items: 1) random sequence generation; 2) allocation concealment; 3) blinding of participants and personnel; 4) blinding of outcome assessments; 5) incomplete outcome data; 6) selection reporting; and 7) other biases. This evaluation was performed independently by Yubing Li and Xinyu Deng. For any disagreement, the results were submitted to a third researcher, Xiao Ma, for resolution.

### 2.5 Mechanism analysis

We summarized the mechanistic pathways in the 13 documents and expanded the possible mechanisms. We also referred to the relevant literature to improve and identify the included mechanism pathways.

### 2.6 Statistical analysis

All outcome indicators were analyzed in Stata 15 because the outcome indicators that we collected were continuous variable data; therefore, the combined-effect size of the standardized mean square difference (SMD) was used for all outcome indicators, with a confidence interval of 95%. Heterogeneity was evaluated using the I-squared (*I* ^*2*^) statistic. When *I* ^*2*^ < 50%, the included studies had good heterogeneity and no source of heterogeneity, and the outcome indicators were analyzed using a fixed-effect model. When *I* ^*2*^ > 50%, heterogeneity was present in the included studies and the outcome indicators were analyzed using a random-effects model. We then applied subgroup analysis to determine the source of the heterogeneity. We used *p-*values to determine significant differences; with *p* < 0.05 indicating a significant difference between the experimental and control groups and *p* > 0.05 indicating no statistically significant difference. In addition, publication bias was determined using Egger’s test, with |t| < 0.05 considered potential publication bias.

## 3 Results

### 3.1 Eligible studies

**FIGURE 1:**
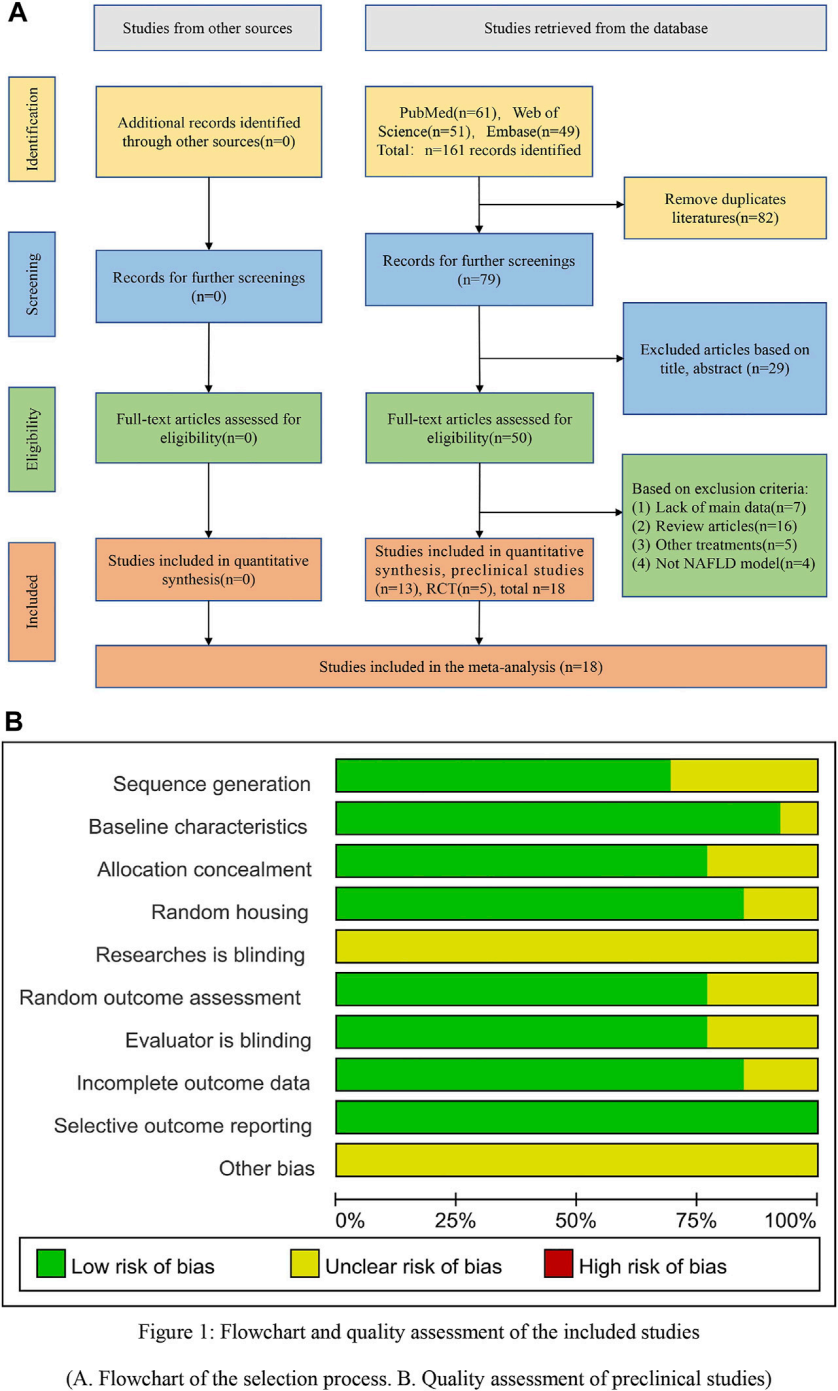
Flowchart and quality assessment of the included studies. **(A)** Flowchart of the selection process. **(B)** Quality assessment of the preclinical studies.

A flowchart showing the literature screening process based on the literature retrieval procedures was constructed (Figure 1A). According to our retrieval strategy, a total of 161 articles were obtained through the preliminary retrieval. Then, 82 duplicated articles and 29 unrelated articles were excluded. Finally, the full text of each result was read, and 13 preclinical and 5 RCT articles met the inclusion criteria.

### 3.2 Characteristics of the included studies

A total of 212 animals were included in 13 articles, including 106 animals in the soybean product group and 106 animals in the model group. The animal species were Sprague–Dawley rats, C57BL/6 mice, and ICR mice. The animal models included high-fat diet induction. Two studies used OLETF rats as a pathological model and reported that soybean did not decrease serum triglycerides (TG) in animals. The administration duration ranged from 4 weeks to 22 weeks. The main reasons were differences in animal species, initial body weight, and drug used. The experimental group was administered different soybean treatments, while the model group was administered normal saline and high-fat diets. The main outcome indicators included TC, TG, ALT, AST, HDL-C, LDL-C, FFA, MDA, SOD, TNF-α, and insulin levels. The therapeutic effects of soybean on non-alcoholic fatty liver were analyzed. Thirteen studies measured TG levels; 13 studies measured TC levels; 6 studies measured AST levels; 7 studies measured ALT levels; 8 studies measured HDL levels; 7 studies measured LDL levels; 8 studies measured FFA levels; 3 studies measured MDA, SOD, and insulin levels; and 5 studies examined TNF-α levels (Supplementary Table S2).

The five RCTs contained a total of 260 patients diagnosed with NAFLD, including 130 patients each in the experimental and control groups, respectively. The patients in the experimental group were treated with soybean, whereas the control group was treated with a placebo. The subjects in the included trials ranged from 43 to 57 years of age. The administration duration was 8 weeks, although one study had a duration of 24 weeks with 8–24 weeks of follow-up. The main outcome indicators included obesity, liver function, and fat indices. Indicators related to insulin resistance, serum insulin, and MDA were also assessed (Supplementary Table S3).

### 3.3 Study quality

The results of the literature quality evaluation showed an average score of 6.6 points (Supplementary Figure S1). Twelve studies reported that the animals were similar at baseline. Nine studies reported that the distribution sequence was fully generated or applied. Ten studies described sufficient animal allocation concealment. Eleven studies reported random assignment of the animals during the experiment. No studies reported that the researchers were blinded during the experiments. Ten studies reported that the animals were randomly selected to evaluate the results, and the evaluators were blinded. Eleven studies reported incomplete data. No studies reported selective results. However, because none of the studies described the other bias, the other bias was not clear (Figure 1B).

RCTs were assessed according to seven aspects of the methodology. The overall quality indicated a relatively low risk of bias, with an average score of 4.4 points (total of 7 points). All RCTs reported selection and reporting biases. Moreover, only one study described allocation concealment, and four studies described the blinding of outcome assessments and performance bias. In addition, three studies reported incomplete outcome data. However, other biases were absent from the included studies (Supplementary Figure S2).

### 3.4 Effects of soybean in preclinical studies

Hematoxylin and eosin (H&E) staining was used in 12 of 13 studies. We carefully read the remaining paper and found that histopathological observation was not performed because it applied a classic OLETF congenital obesity rat model and examined all indicators of non-alcoholic fatty liver disease. Nine studies reported intact liver tissue structure in normal rats, with an orderly arrangement of liver cells, clear nuclear structure, and normal hepatic lobule structure, with no pathological changes such as steatosis and hepatocyte swelling. Rats fed a high-fat diet showed more yellow livers; however, soybean reversed the change in liver color to light brown. Moreover, while HFD feeding induced the accumulation of lipid droplets, an effect significantly reversed by soybean treatment (Panasevich et al., 2017). NAFLD rats showed a significant accumulation of liver fat, as well as inflammatory cell infiltration and liver fibrosis. Micrographs of the liver tissue of NAFLD rats showed laminar and diffuse steatosis. Hepatocytes were noticeably swollen. Most of the nuclei were extruded to one side, and some nuclei were deformed and had disappeared. The results showed that soybean significantly inhibited hepatic steatosis caused by NAFLD and that soybean products decreased the levels of outcome indicators such as TG, TC, AST, ALT, LDL-C, FFA, TNF-α, MDA, and insulin and increased the levels of SOD and HDL-C. Liver function indicators, lipid metabolism indicators, oxidative stress indicators, and inflammatory indicators were individually assessed in the meta-analysis of preclinical studies of soybean for the treatment of NAFLD to investigate the potential of soybean to affect these indicators. The results showed that soybean effectively improved the liver function index, lipid metabolism, and inflammatory factor levels and improved non-alcoholic fatty liver through multiple pathways, including antioxidant stress and lipid metabolism pathways.

#### 3.4.1 Effects on liver function

**FIGURE 2:**
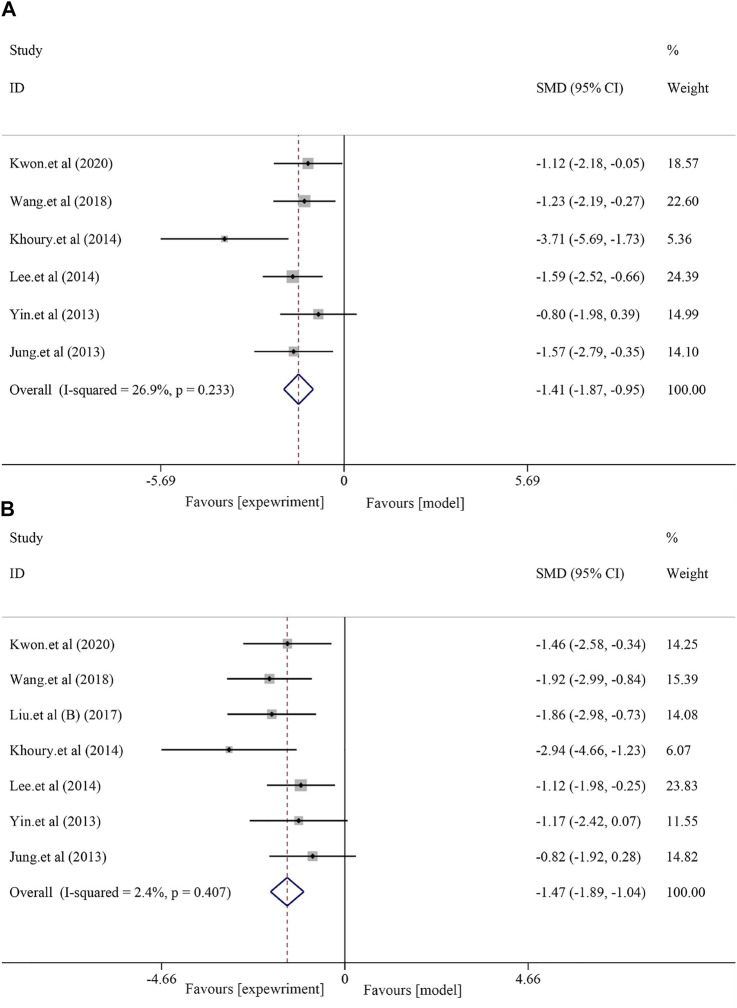
Effect of soybean on AST and ALT levels in preclinical studies. **(A)** Pooled effect of AST. **(B)** Pooled effect of ALT.

A total of six studies with 98 animals examined AST levels after soybean treatment (*I* ^2^ = 26.9% and *p* = 0.233). Due to the minor heterogeneity, a fixed-effect model was used for further analysis. The results showed that soybean significantly reduced the AST level compared to that in the model group [*SMD* = −1.41, *95% CI* (−1.87, −0.95), *p* < 0.0001] (Figure 2A). Seven studies with 116 animals examined ALT levels after soybean treatment. However, the results of the heterogeneity analysis suggested minor heterogeneity (*I* ^2^ = 2.4%, *p* = 0.407). Thus, a fixed-effect model was used. The meta-analysis demonstrated that there was a significant difference between the two groups [*SMD* = −1.47, *95% CI* (−1.89, −1.04), *p* < 0.0001] (Figure 2B).

#### 3.4.2 Effects on fatty liver indicators

**FIGURE 3:**
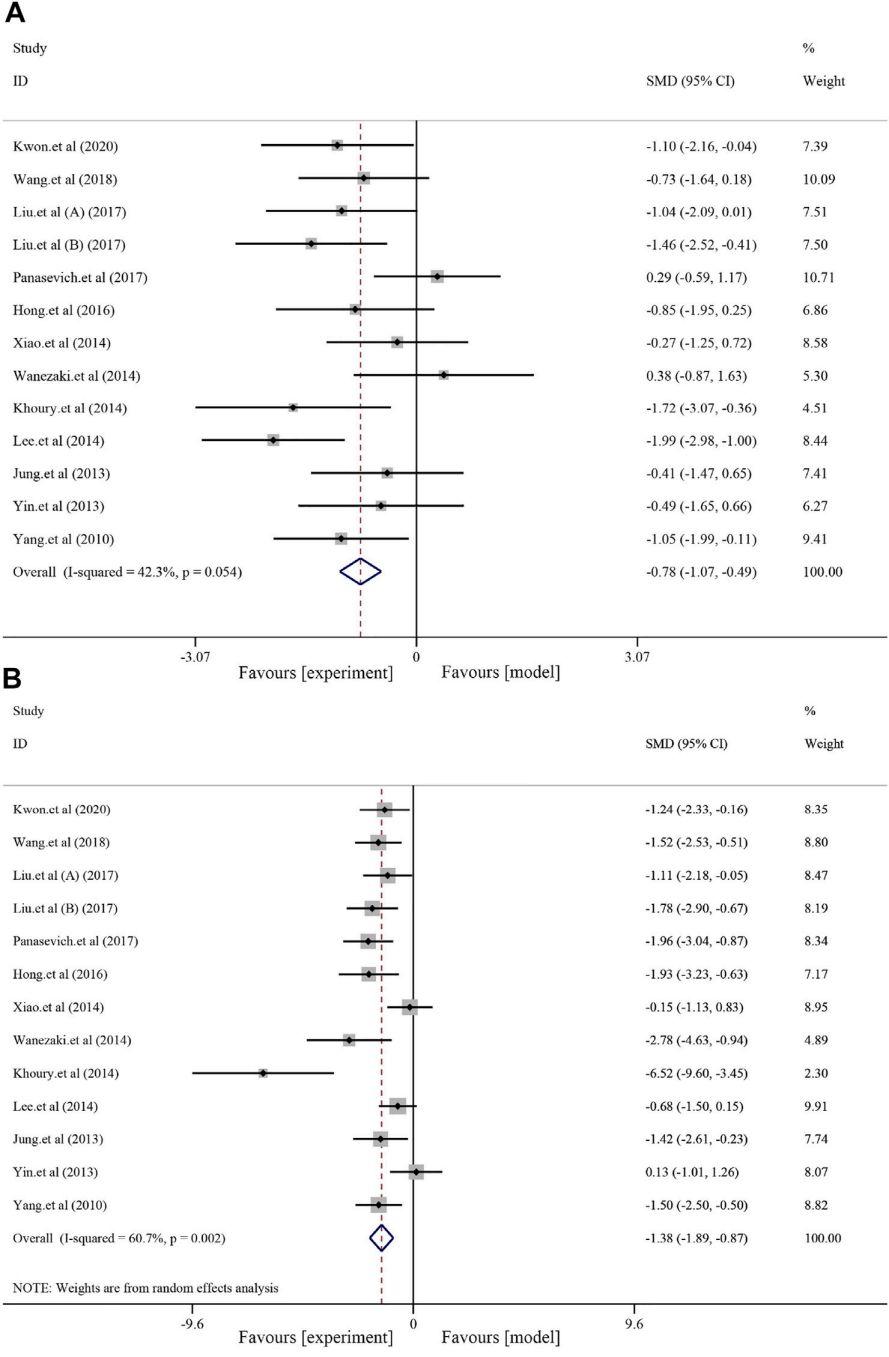
Effects of soybean on TG and TC levels in preclinical studies. **(A)** Pooled effect of TG. **(B)** Pooled effect of TC.

Thirteen studies with 212 animals reported blood TG levels after soybean treatment (*I* ^2^ = 42.3% and *p* = 0.054); thus, a random-effects model was used for further meta-analysis. Compared to that in the model group, soybean reduced blood TG levels [*SMD* = −0.78, *95% CI* (−1.07, −0.49), and *p <* 0.0001] (Figure 3A). A total of 13 studies with 212 animals examined blood TC levels after soybean treatment. Heterogeneity analysis suggested significant heterogeneity (*I* ^2^ = 60.7% and *p* = 0.002); therefore, a random-effects model was used for further analysis. Compared to the model group, soybean significantly reduced blood TC levels [*SMD* = −1.38, *95% CI* (−1.89, −0.87), and *p <* 0.0001] (Figure 3B). Seven studies with 124 animals examined LDL-C levels after soybean treatment for NAFLD. Heterogeneity analysis suggested obvious heterogeneity (*I* ^2^ = 69.8%, *p* = 0.003); therefore, we used a random-effects model in further meta-analyses. The results showed that soybean significantly reduced LDL-C levels [*SMD* = −1.01, *95% CI* (−1.73, −0.29), and *p <* 0.0001] (Supplementary Figure S3A) compared to that in the model group. Eight studies with 138 animals examined HDL levels after soybean treatment for NAFLD. Due to minor heterogeneity [*I* ^2^ = 29.6% and *p* = 0.192], we used a fixed-effect model with a combined effect size. A minor increase in HDL-C level was observed compared to that in the model group [*SMD* = 0.08, *95% CI* (−0.26, 0.42), and *p* = 0.630] (Supplementary Figure S3B), but the differences were not statistically significant. HDL-C is an antiatherosclerotic lipoprotein that is synthesized primarily in the liver and can transport cholesterol from extrahepatic tissue to the liver (Robertson et al., 2004). High serum TC concentrations can stimulate HDL-C transport, leading to transient high cholesterol accumulation in the liver (Yin et al., 2021). Eight studies with 132 animals examined FFA levels after soybean treatment for NAFLD. Due to obvious heterogeneity in FFA levels [*I* ^2^ = 74.7% and *p* < 0.0001], so we used a random-effects model in the meta-analysis. The results indicated that soybean significantly reduced the FFA level compared to that in the model group [*SMD* = −1.55, *95% CI* (−2.38, −0.73), and *p <* 0.000] (Supplementary Figure S4).

#### 3.4.3 Effects on oxidative stress regulation

**FIGURE 4:**
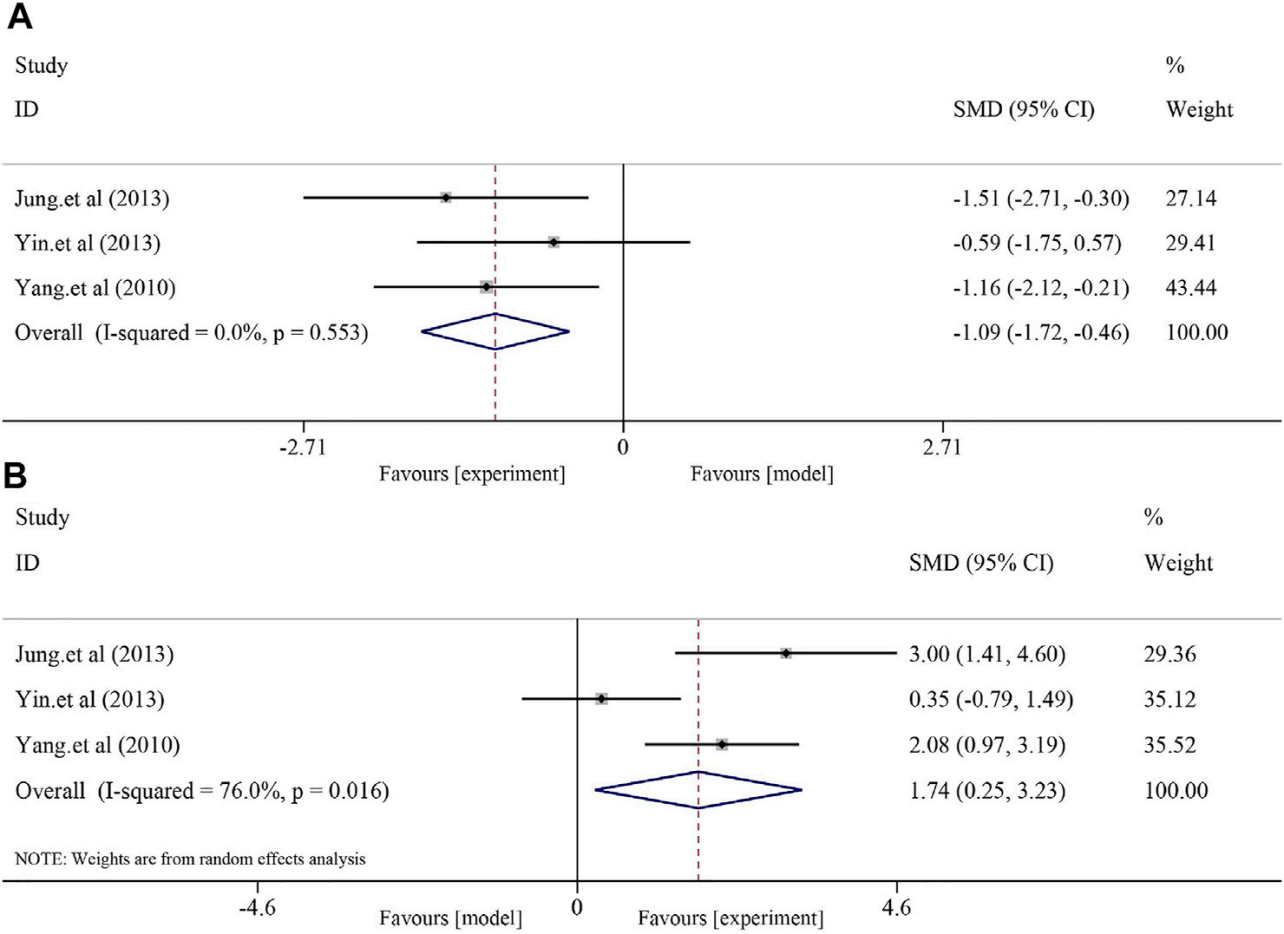
Effect of soybean on MDA and SOD levels in preclinical studies. **(A)** Pooled effect of MDA. **(B)** Pooled effect of SOD.

Soybean significantly reduced MDA levels and increased SOD levels in the NAFLD model. Three studies with 46 animals were included in the analysis of MDA level, and a fixed-effect model was used to combine the effect sizes (*I* ^*2*^ = 0.0% and *p =* 0.553). The results showed that the use of soybean significantly reduced the MDA level compared to that in the model group [*SMD* = −1.09, *95% CI* (−1.72, −0.46), and *p* < 0.0001]. Three studies with 46 animals examined changes in SOD levels. Due to the significant heterogeneity (*I* ^2^ = 76.0% and *p =* 0.016), a random-effect model was used for further meta-analysis. Compared to that in the model group, soybean significantly increased SOD levels [*SMD* = 1.74, *95% CI* (0.25, 3.23), and *p =* 0.022] (Figure 4).

#### 3.4.4 Effects on TNF-α and insulin

We evaluated the anti-inflammatory effects of soybean by comparing TNF-α levels to those in the model group. Five studies with 82 animals examined the level of TNF-α after soybean treatment (*I* ^*2*^ = 7.7% and *p =* 0.363); due to minor heterogeneity, a fixed-effects model was used for further meta-analysis. Soybean significantly reduced TNF-α levels [*SMD* = −1.94, *95% CI* (−2.49, −1.40), and *p* < 0.0001] compared to those in the model group (Supplementary Figure S5A). Three studies with 54 animals examined insulin levels after soybean treatment. However, due to significant heterogeneity (*I* ^2^ = 88.7% and *p <* 0.0001), a random-effects model was used. The meta-analysis revealed a minor difference between the two groups [*SMD* = −0.18, *95% CI* (−1.94,1.58), *p* = 0.842] (Supplementary Figure S5B).

### 3.5 Subgroup analysis of related major indicators

#### 3.5.1 Subgroup analysis of blood TG levels

We performed a subgroup analysis of blood TG in the animal models and among animal species (Supplementary Table S4). The results of the subgroup analysis of the animal models (Supplementary Figure S6) showed that soybean reduced the blood TG levels in the HFD model [n = 182, *SM*D = −0.99, 95% CI (−1.30, −0.67), *p* < 0.0001]. In the OLETF model, soybean had no significant effect on blood TG levels [n = 30, SMD = 0.32, 95% CI (−0.40, 1.04), *p* = 0.383]. The results indicated that soybean significantly reduced the blood TG level in the HFD model but not in the OLETF model. Therefore, we preliminarily concluded that the failure of soybean to reduce blood TG levels may be related to OLETF rats. This may be an unexpected result, which we discuss in a later section. Panasevich et al. (2017) and Wanezaki et al. (2015) used OLETF rats as models and found that soybean did not affect blood TG levels.

The subgroup analysis of mouse species (Supplementary Figure S7) showed that soybean alleviated the blood TG levels in mice [n = 100, SMD = −1.09, 95% CI (−1.53, −0.66), and *p* < 0.0001]. In rat species, although soybean reduced blood TG levels, the change was not statistically significant [Rat, n = 112, SMD = −0.52, *95% CI* (−0.91, −0.13), and *p* = 0.008]. This difference may be related to the use of OLETF rats, which may have affected the analysis results. Therefore, soybean reduced serum TG levels in rats and mice to some extent.

#### 3.5.2 Subgroup analyses of blood TC levels

Thirteen studies with 212 mice examined blood TC levels after soybean treatment for NAFLD. We performed subgroup analyses of blood TC levels in the animal models and among animal species. The results of the subgroup for animal models (Supplementary Figure S8) showed that soybean reduced the blood TC levels in the HFD model [n = 182, SMD = −1.24, 95% CI (−1.78, −0.70), *p* < 0.0001] and in the OLETF model [n = 30, SMD = −2.17, 95% CI (−3.11, −1.24), *p* < 0.0001]. Therefore, soybean significantly reduced the blood TC levels in the HFD and OLETF models.

Subgroup analysis of animal species (Supplementary Figure S9) showed that soybean alleviated the blood TC levels in mice [n = 100, *SM*D = −1.63, 95% CI (−2.46, −0.79), *p* = 0.001] and rats [n = 112, SMD = −1.21, 95% CI (−1.91, −0.52), *p* < 0.0001]. Therefore, soybean reduced the levels of blood TC associated with NAFLD in rats and mice.

### 3.6 Effects of soybean on patients in RCTs

Liver function indicators, anthropometric indices, lipid metabolism indicators, and oxidative stress indicators were individually assessed in the meta-analysis of clinical studies of soybean treatment for NAFLD. The potential for soybean to affect those indicators was investigated. The results showed that soybean improved liver function indices, lipid metabolism, and oxidative stress levels.

#### 3.6.1 Effects on liver function

**FIGURE 5:**
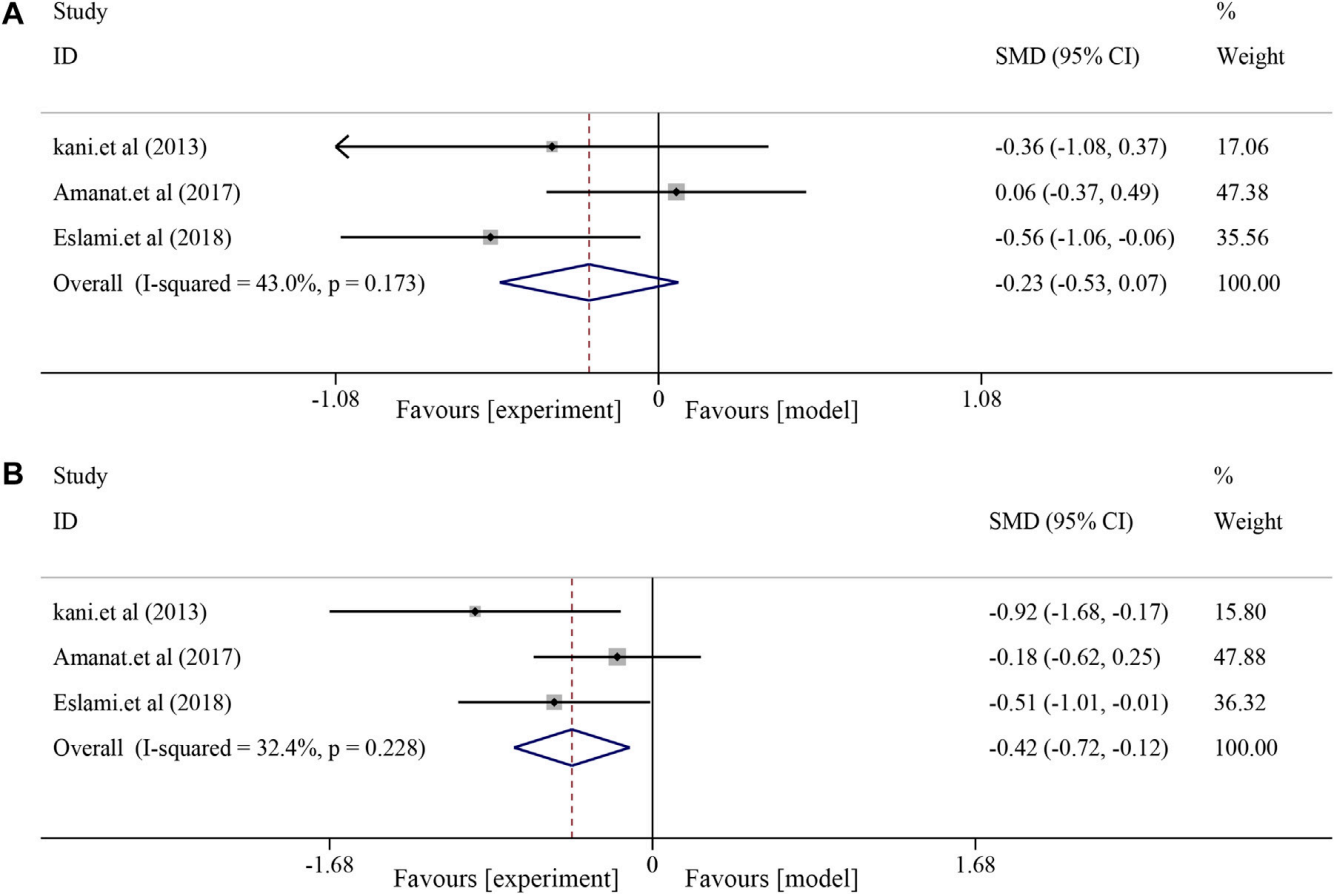
Effects of soybean on AST and ALT levels in clinical studies. **(A)** Pooled effect of AST. **(B)** Pooled effect of ALT.

For liver function indices, a fixed-effects model was used for further analysis due to minor heterogeneity in AST (*I* ^*2*^ = 43.0%, *p* = 0.173) and ALT (*I* ^*2*^ = 32.4%, *p* = 0.228). The results showed no significant effect of soybean on AST levels in patients with NAFLD compared to patients in the control group [*SM*D = −0.23, *95% CI* (−0.53, 0.07), *p* = 0.128] (Figure 5A) and a significant effect on ALT [*SM*D = −0.42, *95% CI* (−0.72, −0.12), *p* = 0.006] (Figure 5B).

#### 3.6.2 Effects on fatty liver indicators

**FIGURE 6:**
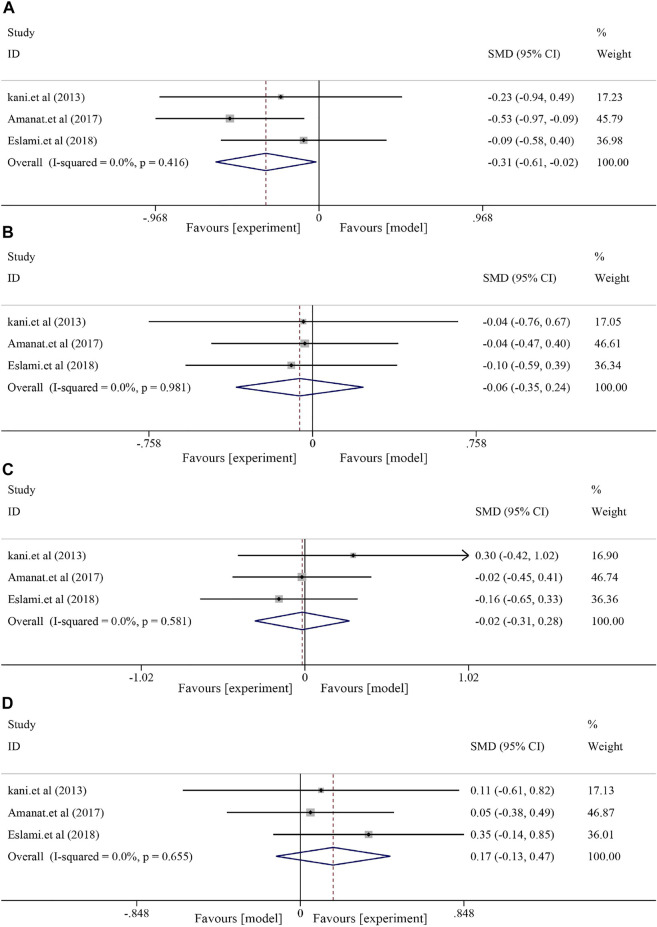
Effect of soybean on TG, TC, LDL, and HDL levels in clinical studies. **(A)** Pooled effects of TG, **(B)** TC, **(C)** LDL, and **(D)** HDL.

The fatty liver indicators containing TG, TC, LDL-C, and HDL-C were compared between the soybean and control groups. The heterogeneities of TG, TC, LDL, and HDL were generally minor (*I* ^*2*^ = 0.0%, *p* = 0.416; *I* ^*2*^ = 0.0%, *p* = 0.981; and *I* ^*2*^ = 0.0%, respectively, *p* = 0.581; *I* ^*2*^ = 0.0%, *p* = 0.655). Soybean use did not significantly decrease serum TG levels [*S*MD = −0.31, 95% CI (−0.61, −0.02), *p* = 0.039] and TC levels [SMD = −0.06, 95% CI (−0.35, 0.24), *p* = 0.694]. Similarly, no significant effect was observed in LDL [SMD = −0.02, 95% CI (−0.31, 0.28), *p* = 0.911] or HDL [SMD = 0.17, 95*% CI* (−0.13, 0.47), *p* = 0.260] (Figure 6).

#### 3.6.3 Effects on anthropometric indices

**FIGURE 7:**
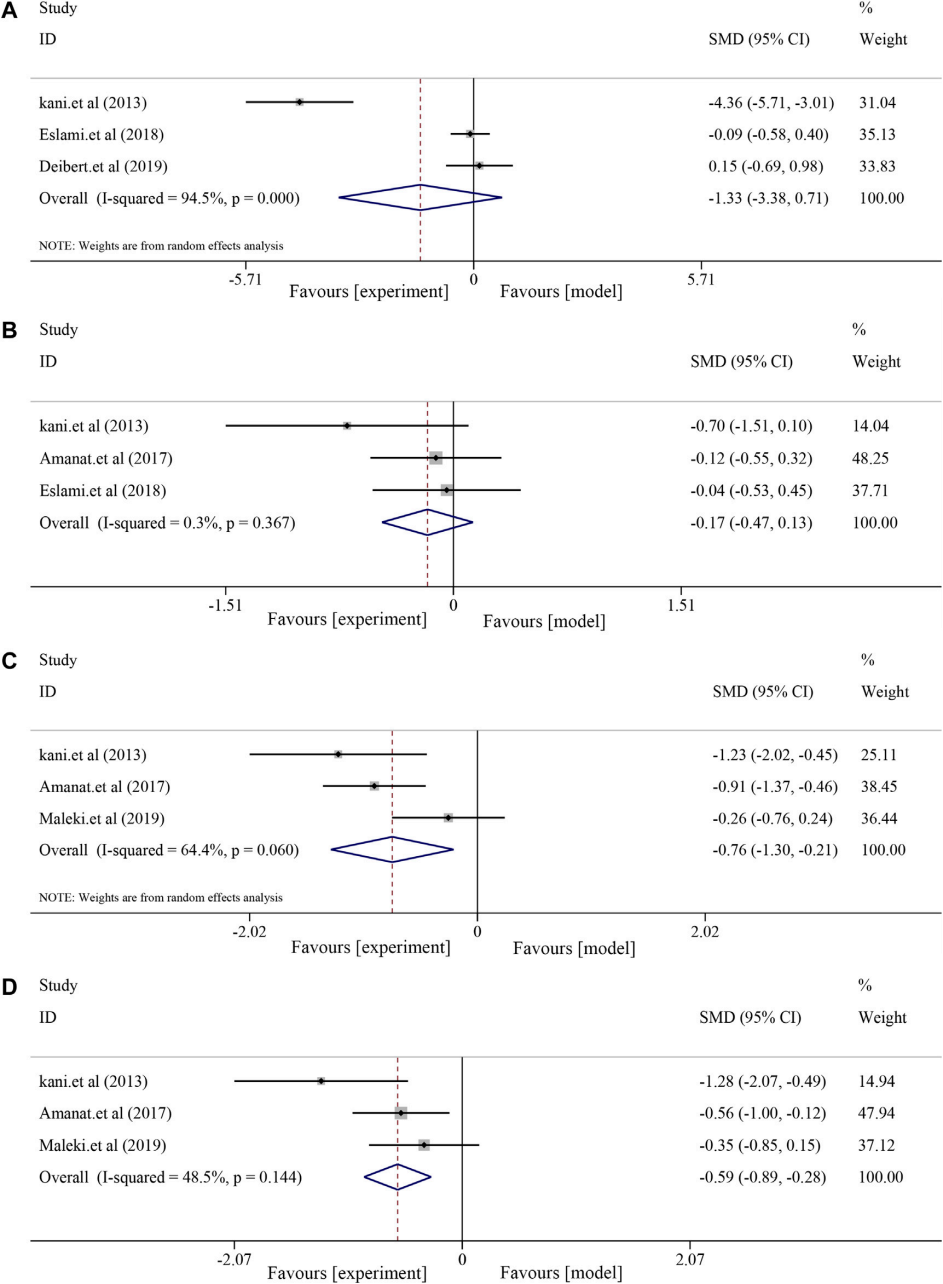
Effects of soybean on BW, BMI, MDA, and insulin levels in clinical studies. **(A)** Pooled effect of BW, **(B)** BMI, **(C)** MDA, and **(D)** insulin.

Three included RCTs were used to assess anthropometric indices, including body weight (BW) and body mass index (BMI). The results showed decreased BW and BMI after soybean treatment, although the differences were not significant [BW, *I* ^*2*^ = 94.5%, *p* < 0.0001, *SMD* = −1.33, *95% CI* (−3.38, 0.71), *p* = 0.202; BMI, *I* ^*2*^ = 0.3%, *p* = 0.367, *SMD* = −0.17, *95% CI* (−0.47, 0.13), *p* = 0.264] compared to the control group (Figure 7).

#### 3.6.4 Effects on MDA and insulin

Analysis of MDA and insulin showed that soybean had a significant effect on MDA [(I2 = 64.6%, *p* = 0.060), SMD = −0.76, 95% CI (−1.30, −0.21), *p* = 0.007] and decreased insulin levels [(I^2^ = 48.5%, *p* = 0.144), SMD = −0.59, *95% CI* (−0.89, −0.28), *p* < 0.0001] (Figure 7).

### 3.7 Publication bias

The main indicators were publication bias (Supplementary Figure S10). The TG outcome of Begg’s test index was *p* = 0.669, the absolute value was >0.05, and the Egger’s test index was *p* = 0.579, which was >0.05, indicating no publication bias. The TC outcome of Begg’s test index was *p* = 0.017 < 0.05, while that of Egger’s test index was *p* = 0.004 < 0.05. The results showed some publication bias in serum TC levels and no significant publication bias in serum TG levels.

### 3.8 Comparison of soybean effects in preclinical studies and clinical trials

We examined a variety of preclinical and clinical indicators to summarize the effect and mechanism of soybean in the treatment of NAFLD. The results strongly suggested that soybean effectively reduced AST and ALT levels to improve NAFLD-induced liver injury and reduce TG, TC, LDL, and FFA and upregulate HDL levels to improve lipid metabolism. In addition, soybean reduced MDA and increased SOD expression levels through oxidative stress pathways, improving NAFLD-induced inflammation by reducing TNF-α levels. Soybean also exerted hepatoprotective effects by improving insulin levels through the insulin resistance pathway. Soybean also showed a therapeutic effect in clinical trials, especially by regulating ALT, TG, insulin, and MDA levels, and showed consistent results in preclinical trials, which may indicate the protective effects of soybean on liver function by improving lipid metabolism, insulin resistance, oxidative stress, and other aspects. In addition, preclinical and clinical studies assessed NAFLD treatment with the soybean extract genistein. The preclinical findings suggested that the continuous administration of 8–64 mg/kg/d of genistein for 12–22 weeks significantly alleviated NAFLD. This finding was consistent with the clinical results; the results of the RCTs demonstrated that the continuous administration of 250 mg/d genistein for 8 weeks was effective in alleviating NAFLD in patients. In the HFD animal model and the OLETF rat model, severe insulin resistance was induced in OLETF rats due to genetic defects. However, soybean improved TG levels in the HFD but not the OLETF model. Patients in clinical trials showed improved TG levels after soybean treatment, which also indirectly demonstrated that soybean could improve lipid metabolism by improving insulin resistance.

### 3.9 Summary of the mechanism of soybean in the treatment of NAFLD

We summarized the mechanistic pathways in the 13 preclinical studies (Supplementary Table S4). The studies showed that soybean products improved non-alcoholic fatty liver disease through lipid metabolism, insulin resistance, inhibiting inflammation, and other mechanisms. Kwon et al. (2020) suggested that soybean affected lipid metabolism in the liver and adipose tissue and promoted the secretion of lipids in adipose tissue to promote the oxidation of liver fatty acids. Jung and Kim (2013) reported that soybean increased SREBP2 levels to suppress ABCA1 expression, increasing cholesterol outflow, reducing cholesterol accumulation in the liver, and improving NAFLD. Khoury et al. (2015) demonstrated that soybean changed the distribution of the T-adjustment factor by using the inherent abilities of the gastrointestinal immune system to induce the T-adjustment factor to inhibit chronic inflammation related to metabolic syndrome, thereby controlling unnecessary systemic immune response. Lee et al. (2014) reported that soybean promoted the expression of fatty factors such as leptin, promoted bile secretion through ApoE, and prevented the accumulation of liver fatty acids, thereby improving NAFLD and providing liver protection. Liu et al. (2017) demonstrated that soybean activated AMPK and regulated the expression of SREBP-1 and PPARα to reduce lipid synthesis and promote fatty acid oxidation through SREBP-1C and PPARα. Panasevich et al. (2017) suggested that soybean improved liver damage by regulating lipid metabolism and intestinal microorganisms. Wanezaki et al. (2015) showed that soybean regulated lipid metabolism in the liver by inhibiting FAS expression or activity and reducing triglyceride levels in the liver. Wang et al. (2018) demonstrated that soybean improved insulin resistance by directly targeting COX-1 and the downstream TXA2 pathway. Yang et al. (2011) suggested that soybean regulated the inflammatory response and immune function to improve oxidative stress, thereby reducing liver cell damage.

## 4 Discussion

**FIGURE 8:**
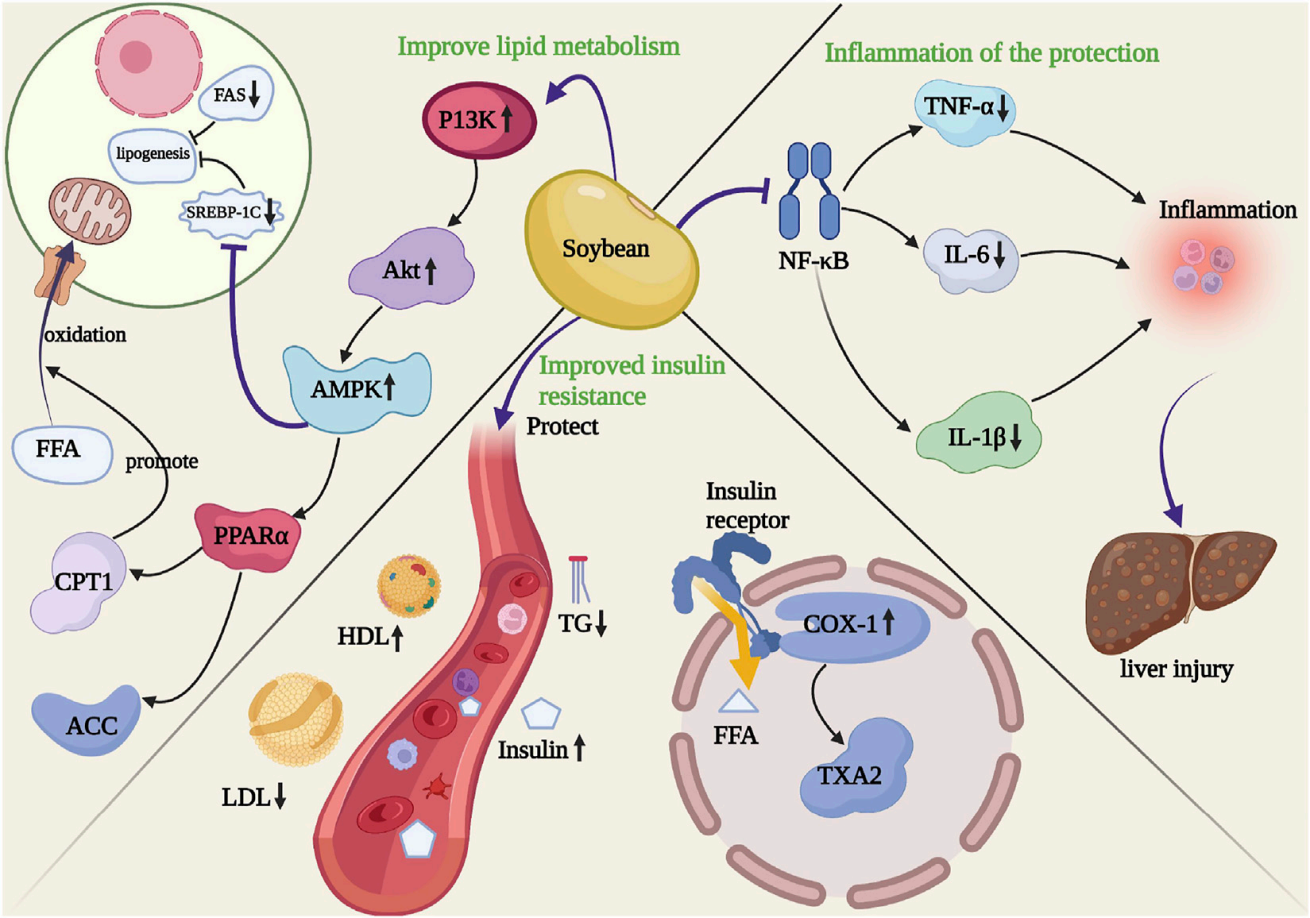
Potential molecular mechanisms for the effects of soybean on NAFLD.

This study systematically evaluated soybean treatment of NAFLD based on preclinical and clinical meta-analyses. Thirteen preclinical studies and five RCTs including 212 animals and 260 patients with non-alcoholic fatty liver disease were collected and analyzed. The results of this study showed that soybean alleviated NAFLD, with significant improvements in liver function and fatty liver indicators. Animals with high-fat diet-induced NAFLD exhibited more yellow liver; however, soybean reversed this change in liver color to dark brown. HFD feeding induced a significant accumulation of lipid droplets, which was reduced with soybean treatment. In addition, the results of this meta-analysis showed that soybean effectively improved lipid metabolism and oxidative stress factors. Analysis of the 13 preclinical studies showed that soybean, which contains mainly isoflavones, decreased TG, TC, AST, ALT, and MDA levels and increased SOD levels. Overall, soybean significantly improved insulin resistance, inhibited inflammation, and regulated lipid metabolic and oxidative stress factors to improve non-alcoholic fatty liver. We also identified the relevant mechanistic pathways (Figure 8).

### 4.1 Regulation of insulin resistance

Soybean did not affect the serum levels of TG in OLETF rats. The pathological manifestations of OLETF rats are insulin resistance and, especially elevated triglyceride levels. Some studies have suggested that serum TG levels are negatively correlated with insulin sensitivity (Yamada et al., 2012). Insulin resistance may cause abnormal triglyceride metabolism through several mechanisms. Insulin is a hormone that regulates adipose tissue and blood flow, significantly reduces blood flow in tissues, and reduces the transport of TG to adipose tissue and skeletal muscle, leading to the accumulation of TG in blood vessels and abnormal lipid metabolism (Shima et al., 1999). Furthermore, insulin resistance increases the release of fatty acids in fat tissue and further increases serum FFA levels. However, increased free fatty acid entry into the liver leads to increased low-density lipoprotein synthesis to increase the endogenous synthesis of TG (Jia et al., 2010). The TXA2 pathway may participate in insulin resistance (Axelsson et al., 2017). The hepatoprotective effect of soybean can be partially explained by the direct targeting of COX-1 and the downstream TXA2 pathway, and some studies have reported that knockout of the *TBXA2R* gene promotes insulin signal transduction (Wang et al., 2018). Thus, we concluded that soybean did not reduce serum TG levels in OLETF rats due to high insulin resistance.

### 4.2 Regulation of lipid metabolism and oxidative stress

NAFLD patients typically have higher TG, TC, and LDL levels and lower HDL levels. TG is oxidized or transported out of the cell by LDL-C and accumulates in the liver once the balance between input and output is disrupted (Liu et al., 2017). Soybean can reduce SREBP-1C and FAS-mediated liposomes and can activate PPARα expression to promote fatty acid oxidation in the liver. Moreover, soybean enhances AMPK phosphorylation. This finding further suggests that soybean products inhibit oxidative stress by activating AMPK. Moreover, SREBP-1c binds to ACC, FAS, and GPAT (Wang et al., 2015) in the target gene promoter region to regulate liver fat production. In patients with NAFLD, liver expression of SREBP-1C and related fatal genes increases, which may be one explanation for increased lipid production in the liver (Yokozawa et al., 2012) and increased HFD-induced SREBP-1c protein and FAS and GPAT mRNA levels. After soybean treatment, the levels of these fat-generation factors were significantly reduced. Thus, soybean may reduce SREBP1-c and inhibit lipid peroxidation, thereby reducing liver fat degeneration.

Soybean activates AMPK through P13K/AKT, which inhibits the expression of liver sterol regulatory element-binding protein (SREBP-1C) and activates peroxidase (PPARα). AMPK is a “stress indicator” that regulates liver lipid metabolism. AMPK activation results in the inactivation of ACC (Winder and Hardie, 1996), which is a key enzyme that catalyzes malonyl-CoA production, thereby enhancing CPT-1 activity. AMPK/PPARα activation promotes CPT1-mediated fatty acid transport into the mitochondria for β-oxidation (Day et al., 2017). Some studies have suggested that soybean increases the protein expression of PGC1α, ACOX, and PPARα to stimulate the β-oxidation of mitochondrial and peroxisomal fatty acids, thereby increasing the catabolic effect of FFAs and preventing adipogenesis and lipid accumulation. Thus, AMPK/PPARα activation may be an important mechanism by which soybean inhibits lipid accumulation.

PPARα is an important regulatory factor that affects lipid metabolism, plays an important role in the liver, and can directly regulate the expression of target genes that participate in fatty acid oxidation, such as CPT-1 and ACO (Pawlak et al., 2015). PPARα significantly improves NAFLD-related symptoms and slows the degree of liver fat damage, thereby delaying NAFLD progression. Staels et al. (2013) reported that PPARα agonists protected against liver fat degeneration. PPARα activation (Mittelman et al., 2002) increases the oxidation of fatty acids and the expression of the fatty acid oxidation genes, reducing synthetic fatty acids and topical fatty acids, thereby reducing lipid deposition. PPARα can also directly improve liver fatty protein and lipid metabolism genes, and the β-oxidation of liver FFAs increases to inhibit lipid deposition in the liver (Schoonjans et al., 1996). PPARα regulates the encoding of the fatty acid transporter transporters 1 (FATP1) gene and promotes fatty acid absorption (Poirier et al., 2001). PPARα activation can regulate fatty acid dial enzymes (FAT/CD36), increasing fatty acid transfer (Motojima et al., 1998). Studies have shown that PPARα-knockout mice can have severe fat infiltration in hepatic tissue and significantly higher blood TG levels compared to those in normal mice. Therefore, inhibiting PPARα expression can cause lipid deposition in the liver and accelerate fatty liver occurrence and development (Neuschwander-Tetri and Caldwell, 2003). Soybean can effectively reverse changes in PPARα, CPT-1, and ACO mRNA levels caused by high-fat treatment, suggesting that soybean can regulate SREBP-1 and PPARα expression and lipid metabolism, which may be related to AMPK activation. Additionally, the accumulation of FFAs in hepatocytes promotes mitochondrial β-oxidation. Overburdening this metabolic pathway leads to an imbalance in fatty acid metabolism and can cause mitochondrial damage, increased oxidative stress, and steatosis. ROS formation induces lipid peroxidation, which alters mitochondrial DNA. Furthermore, high-sugar or high-cholesterol diets can cause excessive infiltration of cholesterol in the mitochondrial membrane, leading to mitochondrial damage, thus causing oxidative stress. These results showed that soybean can regulate the protein expression of PGC-1α and CYP2E1 to decrease ROS and MDA production, resulting in improved mitochondrial function. Therefore, soybean can improve oxidative stress and maintain mitochondrial homeostasis by inhibiting fat synthesis and reducing fat accumulation. The integrity of mitochondrial function further promotes the β-oxidation of free fatty acids and improves lipid metabolism. However, the levels of FFAs (*SM*D = −1.55, *p* < 0.000) and MDA (SMD = −1.09, *p* < 0.0001) decreased significantly, while that of SOD increased significantly after soybean treatment (SMD = 1.74, *p* = 0.022) in preclinical studies. These results suggested that soybean inhibited fat synthesis and promoted fat decomposition by improving lipid metabolism and maintaining the integrity of mitochondrial function by improving oxidative stress. Thus, β-oxidation was promoted, and fat accumulation was further reduced.

### 4.3 Regulation of inflammation

The liver is an important organ that regulates body lipid metabolism through the intake of FFAs and the synthesis, storage, and output of lipids (Rui, 2014). The liver transfer cycle of FFAs can impair the insulin sensitivity of this organ (Cusi, 2012), induce SREBP-1C transcription, and lead to liver fat regeneration. Soybean regulates the hepatic NF-κB pathway and Kupffer cells by inhibiting TNF-α, IL-6, and IL-1β generation (Boden et al., 2005; Parker, 2018). Decreasing TNF-α levels can improve the insulin sensitivity of adipocytes, and the consequent decreased release of FFAs can alleviate liver burden (Kern et al., 2001; Zhang et al., 2002). Therefore, soybean improves insulin resistance by improving inflammation, thereby protecting the liver. In this study, the normalization of serum FFA levels after treatment with soybean was explained by decreases in fat production, inflammation, and hepatocyte damage (Kwon et al., 2020). The use of soybean-related products improves hepatic fat degeneration. The cellular mechanisms underlying these improvements have been reported (Wang et al., 2017).

### 4.4 Limitations

1) Although the effectiveness of soybean in the treatment of NAFLD has been established by examining pathological indicators, the main components that result in the treatment of NAFLD have not been fully determined due to the multiple components in soybean. Thus, further studies are needed to investigate the optimal ingredient for the treatment of NAFLD. 2) In addition, the administration of therapeutic drugs mixed with a high-fat diet cannot be used to determine the specific dose. Therefore, subsequent studies should focus on the specified dose of therapeutic drugs to provide a research basis for clinical studies. 3) The results of the meta-analysis showed some heterogeneity. This heterogeneity was influenced by a combination of factors such as different laboratories using different instruments to determine the active components of soybeans, the duration of administration, the dose administered, and the soybean extraction process. 4) Among the 18 articles included in this study, none described blinding during the experiment, which may lead to selection and implementation biases.

### 4.5 Implications

Many animals are used for scientific research annually, and the worldwide standards concerning the use of laboratory animals have also become more rigorous. Therefore, the effective use of animal models and research results is an important means to reduce unnecessary animal experiments. A meta-analysis of animal experiments can make full use of animal resources and reduce unnecessary sacrifice of experimental animals, which is more ethical. Our study provides evidence for the research of natural drugs, such as natural herbal and dietary ingredients, and provides examples of methods for the meta-analysis of animal studies. The effect of soybean varies depending on the animal model and the time of administration. Therefore, in an experimental design, different models and administration times are used depending on the experimental purpose. The selection of an appropriate animal model must consider the differences between diseased organs in animals and humans. In addition, the dose range of soybean requires more attention. Therefore, subsequent studies should focus on the dose of therapeutic agents to provide a research basis for clinical studies.

## 5 Conclusion

The preclinical results showed that animals treated with soybean showed significant improvements. Compared to the model group, soybean effectively reduced major outcome indicators in the experimental group, including AST, ALT, TG, TC, HDL, LDL, MDA, TNF-α, and insulin levels, and increased FFA and SOD levels. The clinical results also indicated the hepatoprotective effect of soybean, especially regarding ALT, TG, MDA, and insulin levels, results that were consistent with those of the preclinical studies. Mechanistically, soybean can significantly ameliorate lipid metabolism and oxidative stress, improve insulin resistance, suppress inflammation, and improve liver function. Therefore, these findings support soybean as a therapeutic agent to prevent and improve NAFLD; these findings also provide a theoretical basis for exploiting NAFLD drugs through evidence-based medical means.

## Data Availability

The original contributions presented in the study are included in the article/Supplementary Material. Further inquiries can be directed to the corresponding authors.
