# Supplementary material for: Preclinical and clinical evidence for the treatment of non-alcoholic fatty liver disease with soybean: A systematic review and meta-analysis
Source: Front Pharmacol. 2023 Jan 25;14:1088614. doi: 10.3389/fphar.2023.1088614 (PMC9907442; doi:10.3389/fphar.2023.1088614)
Supplement: Supplementary file 1 [file Table1.docx]

**Catalog of figures and tables in supplementary materials**

**Supplementary Tables**

Supplementary Table 1. Literature search strategy for soybean in the treatment of NALFD

Supplementary Table 2. Characteristics of preclinical studies

Supplementary Table 3. Characteristics of clinical trials

Supplementary Table 4. Possible mechanism of soybean on NAFLD

Supplementary Table 5. Abbreviations

**Supplementary Figures**

Supplementary Figure 1. The risk of bias graph of included studies.

Supplementary Figure 2 Methodological quality assessment of clinical trials

Supplementary Figure 3 Effect of soybean on LDL-C and HDL-C level in preclinical studies

Supplementary Figure 4. Effect of soybean on FFA level in preclinical studies

Supplementary Figure 5. Effect of soybean on TNF-α and Insulin level in preclinical studies

Supplementary Figure 6. Subgroup analysis of TG based on animal model

Supplementary Figure 7. Subgroup analysis of TG based on animal species

Supplementary Figure 8. Subgroup analysis of TC based on animal model

Supplementary Figure 9. Subgroup analysis of TC based on animal species

Supplementary Figure 10. Egger’s test for TG and TC in preclinical studies

Supplementary Table 1. Literature search strategy for soy in the treatment of NALFD

| **Search Strategy (Pubmed)** | |
| --- | --- |
| #1 | soybean [All Fields] |
| #2 | soya bean [All Fields] |
| #3 | soy [All Fields] |
| #4 | #1or#2or#3 |
| #5 | Non-alcoholic fatty liver disease [MeSH Terms] |
| #6 | NAFLD [MeSH Terms] |
| #7 | NASH [MeSH Terms] |
| #8 | #5 or #6 or #7 |
| #9 | #4and#8 |

**Supplementary Table 2. Characteristics of preclinical studies**

| Study  (year) | Species | Model | soybean group | Positive control group | duration | Outcome index |
| --- | --- | --- | --- | --- | --- | --- |
| Kwon.et al (2020) | Male C57BL/6J mice | HFD | Soybean group: Germinated Soybean Embryo Extract (45 mg/kg), which contains soyasaponin Ab (77.0 ± 1.3 mg/g)  Model group: 10% fat diet | No reported | 10 Weeks | TG,TC,AST,ALT,  LDL,HDL,FFA,TNF-α |
| Wang.et al (2018) | Male C57BL/6J mice | HFD | Soybean group: genistein (64 mg/kg)  Model group: High fat diet | aspirin (120mg/kg) | 22 Weeks | TG,TC,AST,ALT,TNF-α,Insulin |
| Liu.et al (A) (2017) | Male SD Rat | HFD | Soybean group: genistein (8 mg/kg)  Model group: High fat diet (60% fat energy) | No reported | 12 Weeks | TG,TC,LDL,HDL,FFA |
| Liu.et al (B) (2017) | Male SD Rat | HFD | Soybean group: soy isoflavone (20 mg/kg)  Model group: High fat diet (60% fat energy) | No reported | 12 Weeks | TG,TC,ALT,FFA |
| Panasevich.et al (2017) | Male OLETF rat | OLETF | Soybean group: soy protein isolate (Daidzein, genistein and glycitein content of the SPI was 453, 731 and 62 μg/g protein) with high-fat diet.  Model group: a semipurified diet containing 23 % protein, 49 % carbohydrate and 17 % fat ad libitum | No reported | 16 Weeks | TG,TC,LDL,HDL,FFA, Insulin |
| Hong.et al (2016) | Male C57BL/6J mice | HFD | Soybean group: 20% soybean embryo powder mixed with high-fat diet  Model group: high-cholesterol and high-fat diet | No reported | 10 Weeks | TG,TC,HDL,FFA, TNF-α |
| Wanezaki.et al (2015) | Male OLETF rat | OLETF | Soybean group: 9.95% (w/w) Soy β-conglycinin mixed with high-fat diet  Model group: high-cholesterol and high-fat diet | No reported | 4 Weeks | TG,TC |
| Khoury.et al (2015) | Mice | HFD | Soybean group: soy extracts 3 μg MI- fraction contains mainly isoflavones, sugars, and phosphatides, phytosterols, saponins).  Model group: high-cholesterol and high-fat diet | No reported | 12 Weeks | TG,TC,AST,ALT, TNF-α |
| Xiao.et al (2014) | Male SD Rat | HFD | Soybean group: soy isoflavone (50 mg/kg diet) mixed with high-fat diet  Model group: high-fat diet containing 20 % casein | No reported | 90 Days | TG,TC,LDL,HDL,FFA |
| Lee.et al (2014) | Male C57BL/6J mice | HFD | Soybean group: soy phosphatidylcholine 2.5 g/kg/d with high-fat diet  Model group: high-fat diet | No reported | 8 Weeks | TG,TC,AST,ALT,LDL,HDL |
| Jung.et al (2013) | Male ICR mice | HFD | Soybean group: 4% black soybean powder mixed with high-fat diet  Model group: high cholesterol and high fat diet | No reported | 12 Weeks | TG,TC,AST,ALT,MDA,  SOD,Insulin |
| Yin.et al (2013) | Male SD Rat | HFD | Soybean group: 2% soy phospholipids mixed with high-fat diet  Model group: high fat diet | No reported | 4 Weeks | TG,TC,AST,ALT,LDL,  HDL,FFA,MDA,SOD |
| Yang.et al (2010) | Male SD Rat | HFD | Soybean group: 11% soy protein isolate mixed with high-fat diet  Model group: high-fat liquid diet | No reported | 6 Weeks | TG,TC,HDL,LDL,  FFA, TNF-α,MDA,SOD |

**Supplementary Table 3. Characteristics of clinical trials**

| Study (year) | Population | N (T/C) | Intervention of experimental group | Intervention of  control group | Duration | Outcome index |
| --- | --- | --- | --- | --- | --- | --- |
| Kani (2013) | Patients with NAFLD | 30(15/15) | Low calorie low carbohydrate diet with soybean nut 30g/d | Low calorie low carbohydrate diet | 8W | BW,BMI,ALT,AST,TG,TC,LDL,HDL,MDA |
| Amanat (2017) | Patients with NAFLD | 82(41/41) | Genistein 250 mg/d | Placebo | 8W | BW,BMI,WC, ALT,AST,Insulin,HOMA-IR,  TG,TC,LDL,HDL |
| Eslami (2018) | Patients with NAFLD | 64(32/32) | 240 ml of soybean milk per day | low-calorie diet | 8W | BW,BMI,WC,ALT,AST,Insulin, TG,TC,LDL,HDL |
| Maleki (2019) | Patients with NAFLD | 62(31/31) | 240 ml of soybean milk per day | low-calorie diet | 8W | BW,BMI, Insulin,  HOMA-IR,MDA |
| Deibert (2019) | Patients with NAFLD | 22(11/11) | Soy protein isolate 66 g/d within the first six weeks, then 33 g/d | Lifestyle change | 24W | BW,BMI,WC,ALT,AST,TG,TC,LDL,HDL, Glucose |

**Supplementary Table 4. Possible mechanism of Soy on NAFLD**

| Study | Animal model | Intervention | Targets/pathways/mechanisms |
| --- | --- | --- | --- |
| Kwon.et al (2020) | HFD | Germinated Soybean Embryo Extract | Upregulation: mTORC1/Akt signaling pathway, PPARα, FFA  Downregulation: SREBP1c |
| Wang.et al (2018) | HFD | Genistein  64 mg/kg | Upregulation: improve insulin resistance  Inhibition: COX-1/ TXA2 signaling pathway  Downregulation: PPAR-γ, and SREBP1 |
| Liu.et al (A) (2017) | HFD | Genistein  8 mg/kg | Upregulation: AMPK, PPARα, CPT-1  Downregulation: SREBP-1c, FAS |
| Liu.et al (B) (2017) | HFD | Soy isoflavone  20 mg/kg | Upregulation: PPARα  Downregulation: SREBP-1c, FAS |
| Panasevich.et al (2017) | OLETF | Soy protein isolate | Upregulation: Fgfr4 and Fxr, Zo1  Improve: lipid metabolism, bile acid metabolism, gut microbiota |
| Hong.et al (2016) | HFD | Soybean Embryo | Upregulation: AMPK, PPARα  Downregulation: FFA, SREBP-1c, FAS |
| Wanezaki.et al (2015) | OLETF | Soy β-conglycinin | Upregulation: CPT-1  Downregulation: FAS, ACC1 |
| Khoury.et al (2015) | HFD | Soy-extracts exerts | Upregulation: AMPK, PPARα  Improve: Insulin resistance  Downregulation: TNF-α |
| Xiao.et al (2014) | HFD | Soy isoflavone  50 mg/kg | Downregulation: PPARγ2, FSP27 |
| Lee.et al (2014) | HFD | Soy Phospholipids | Downregulation: ApoE, Leptin |
| Jung.et al (2013) | HFD | Black soybean | Upregulation: AMPK, PPARα, ABCA1  Upregulation: HMG-CoA, SREBP-1c |
| Yin.et al (2013) | HFD | Soy Phospholipids | Upregulation: docosahexaenoic acid,  Downregulation: docosahexaenoic acid, MDA, TBA, CRP |
| Yang.et al (2010) | HFD | Soy protein | Downregulation: CYP2E1, TNF-α |

| **Supplementary Table 5. Abbreviations** | |
| --- | --- |
| **Abbreviation** | **Full Name** |
| SMD | Standardized Mean Difference |
| 95%CI | 95% Confidence Interval |
| I^2^ | I-square |
| TG | triglycerides |
| TC | total cholesterol |
| LDL-C | low-density lipoprotein cholesterol |
| HDL-C | high-density lipoprotein cholesterol |
| ALT | alanine aminotransferase |
| AST | aspartate aminotransferase |
| FFA | Free fatty acid |
| CAT | catalase |
| TNF-α | tumor necrosis factor-α |
| TGF-β1 | transforming growth factor-β1 |
| IL-1β | interleukin 1 beta |
| PGC‐1α | Peroxisome proliferator-activated receptor-gamma co-activator-1alpha |
| Nrf2 | Nuclear factor erythroid 2-related factor 2 |
| AMPK | Activated protein kinase |
| PI3K | Identification of the phosphoinositide-3-kinase |
| SREBP2 | Sterol regulatory element-binding protein 2 |
| NF-κB | Nuclear factor-kappaB |
| HFD | High-fat diet |
| NAFLD | Non-alcoholic fatty liver disease |
| NASH | Non-alcoholic steatohepatitis |
| COX-1 | cyclooxygenase-1 |
| TXA2 | Thromboxane A2 |
| OLETF | Otsuka Long-Evans Tokushima Fatty |
| PPARα | peroxisome proliferators-activated receptors α |
| ACC | Acetyl-CoA carboxylase |
| FAS | Fatty acid synthase |
| FATP1 | fatty acid transporter transporters 1 |
| CPT1 | carnitine palmitoyltransferase-1 |
| ACO | Acyl-CoA Oxidase |


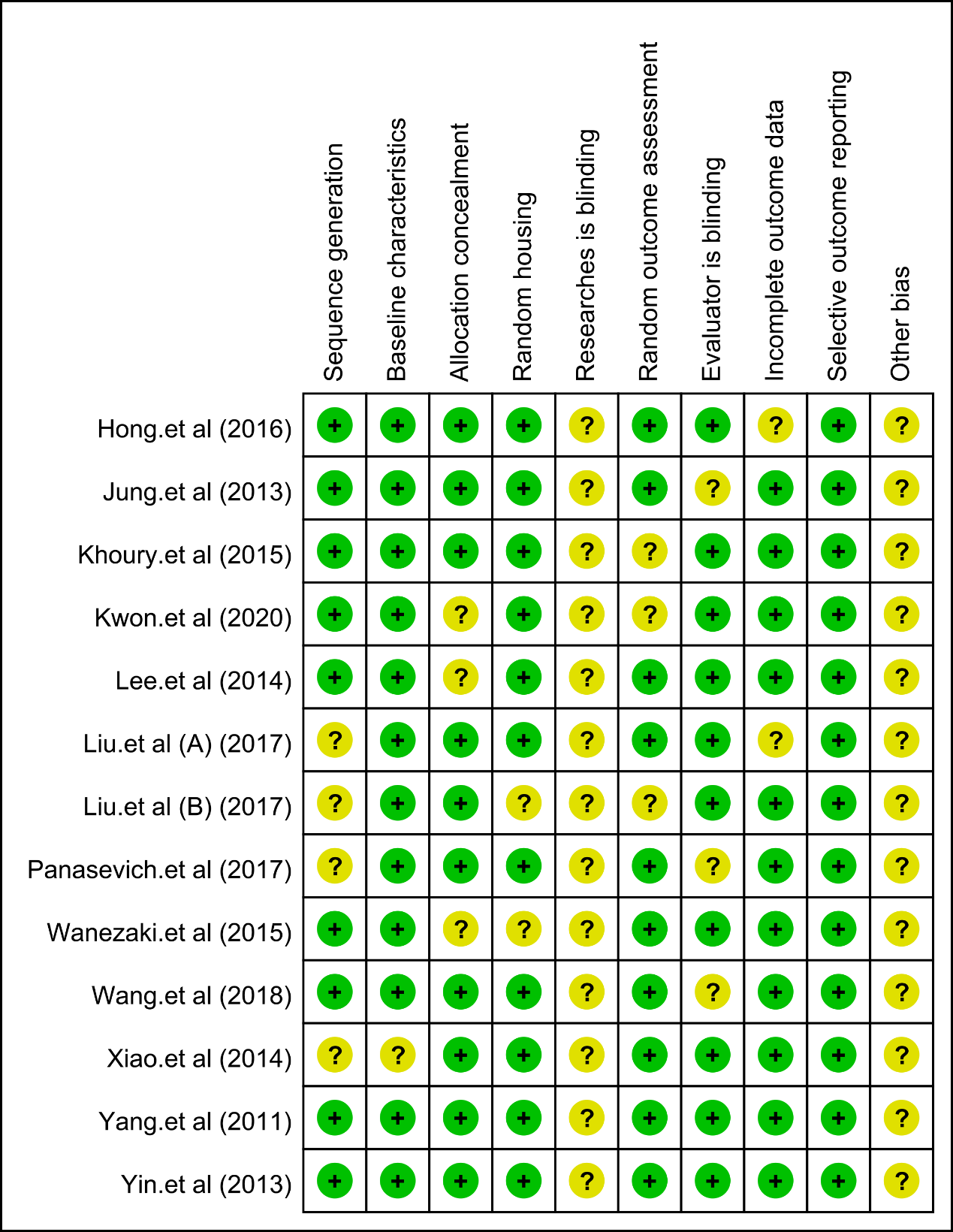


**Supplementary Figure 1. The risk of bias graph of included studies.**


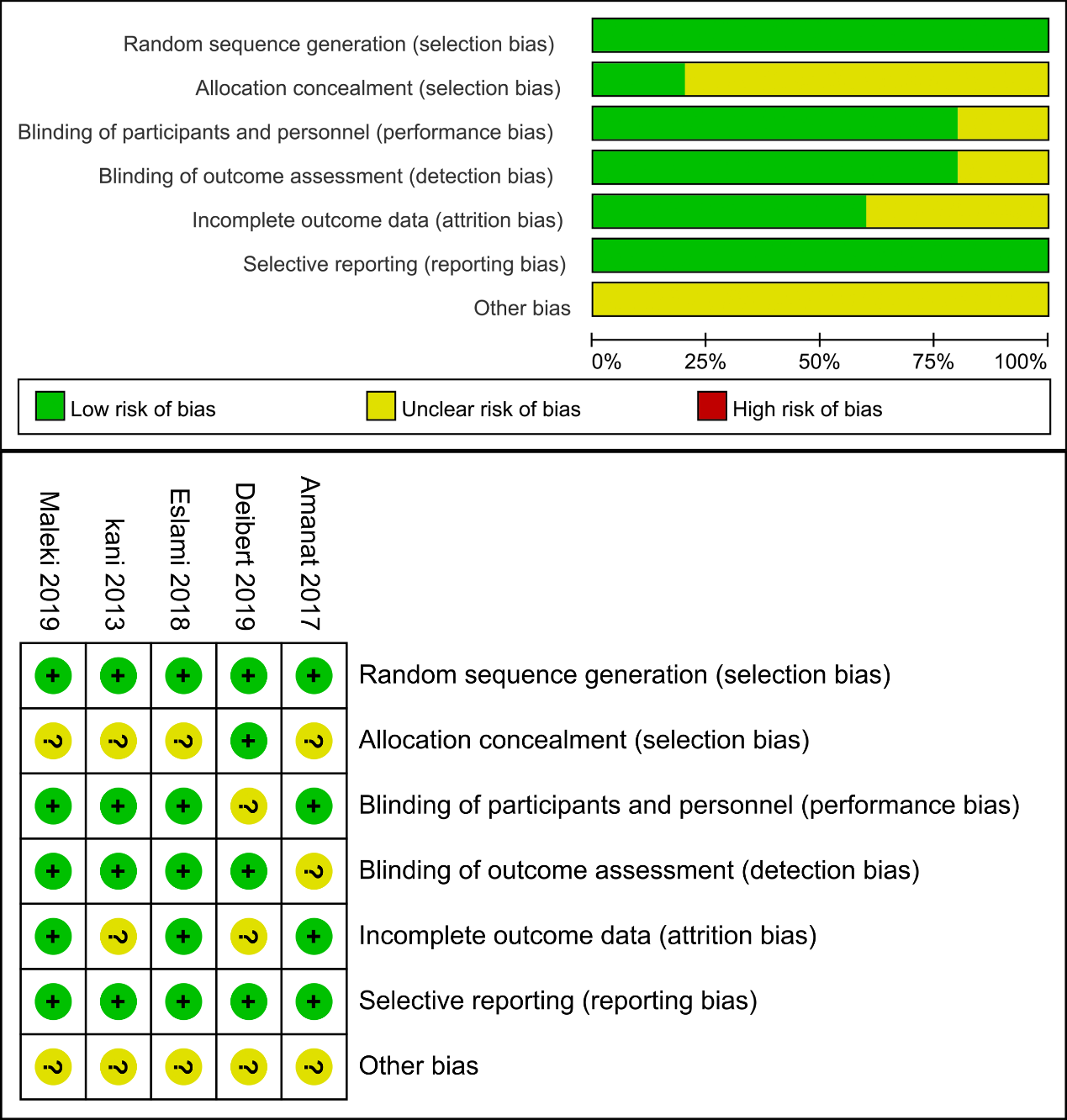


**Supplementary Figure 2** **Methodological quality assessment of clinical trials**


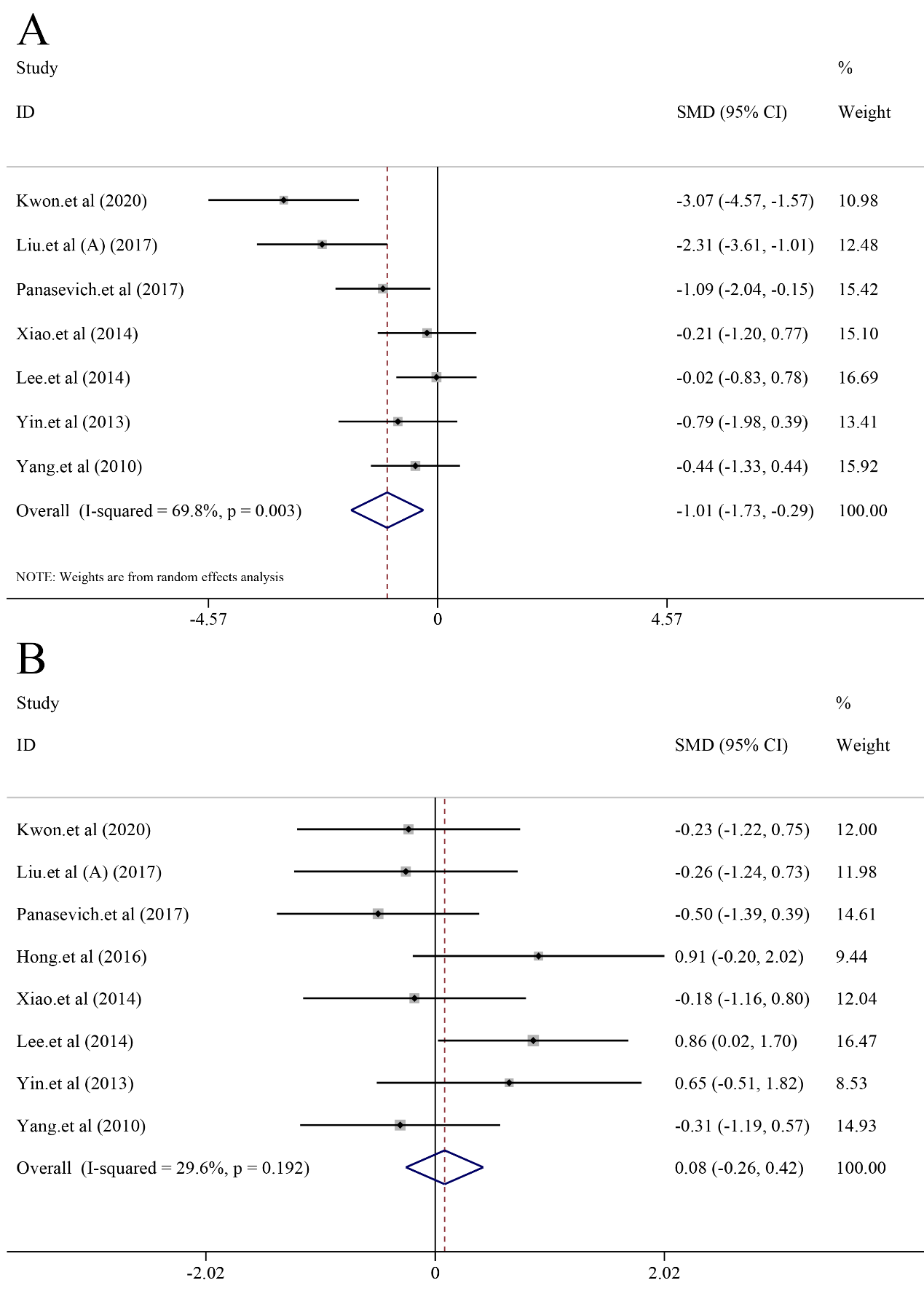


**Supplementary Figure 3 Effect of soybean on LDL-C and HDL-C level in preclinical studies**

(A. Pooled effect of LDL-C; B. Pooled effect of HDL-C)


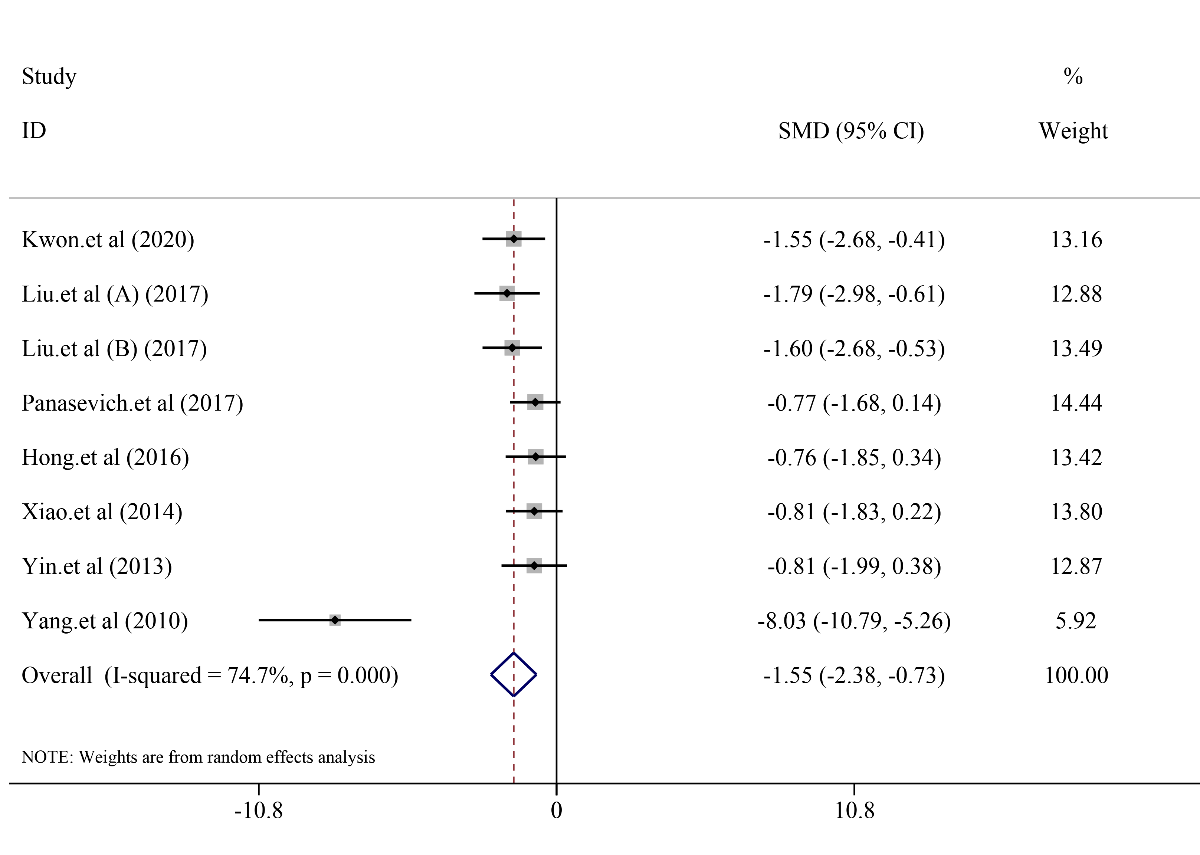


**Supplementary Figure 4. Effect of soybean on FFA level in preclinical studies
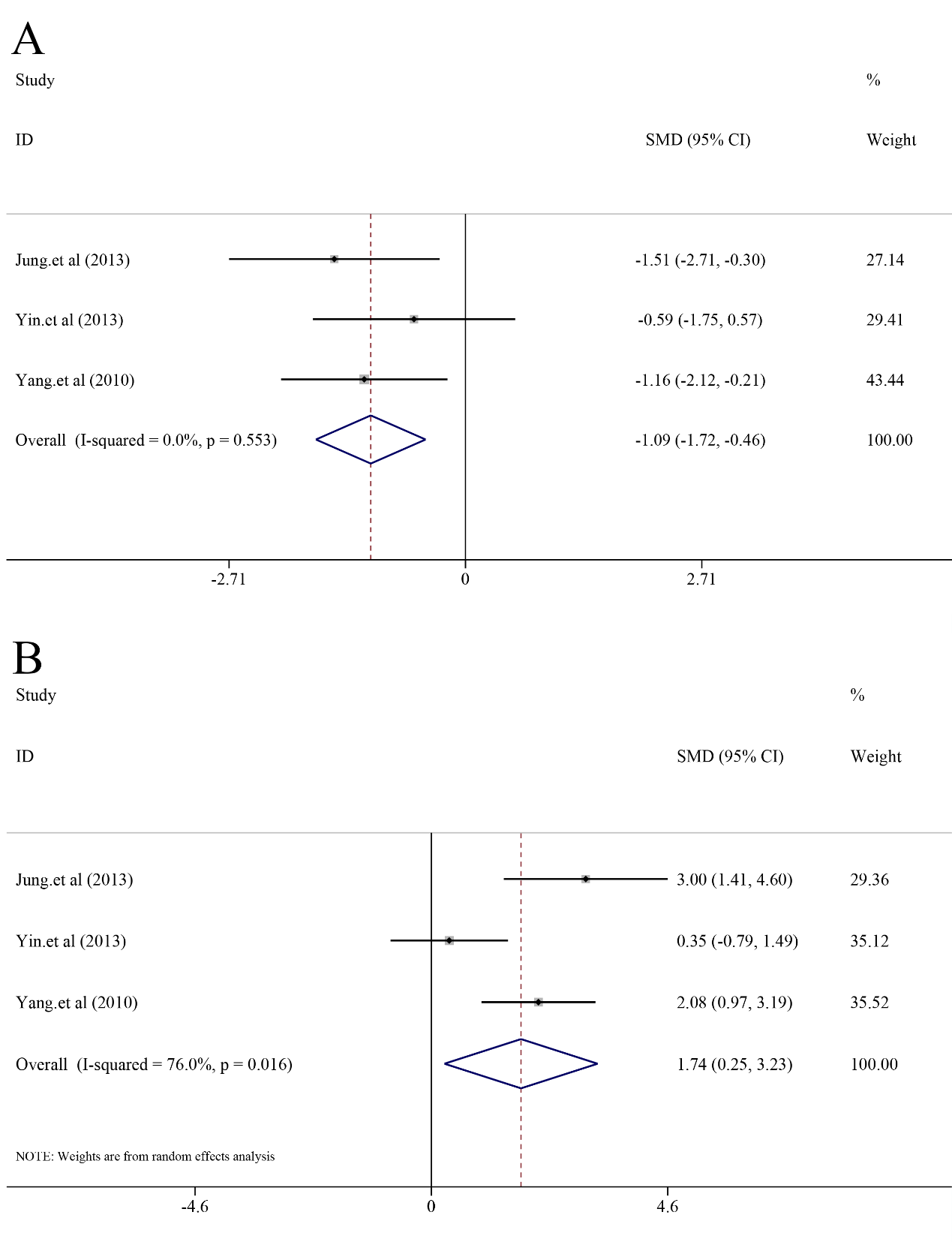
**

**Supplementary Figure 5. Effect of soybean on MDA and SOD level in preclinical studies**

(A. Pooled effect of MDA; B. Pooled effect of SOD)


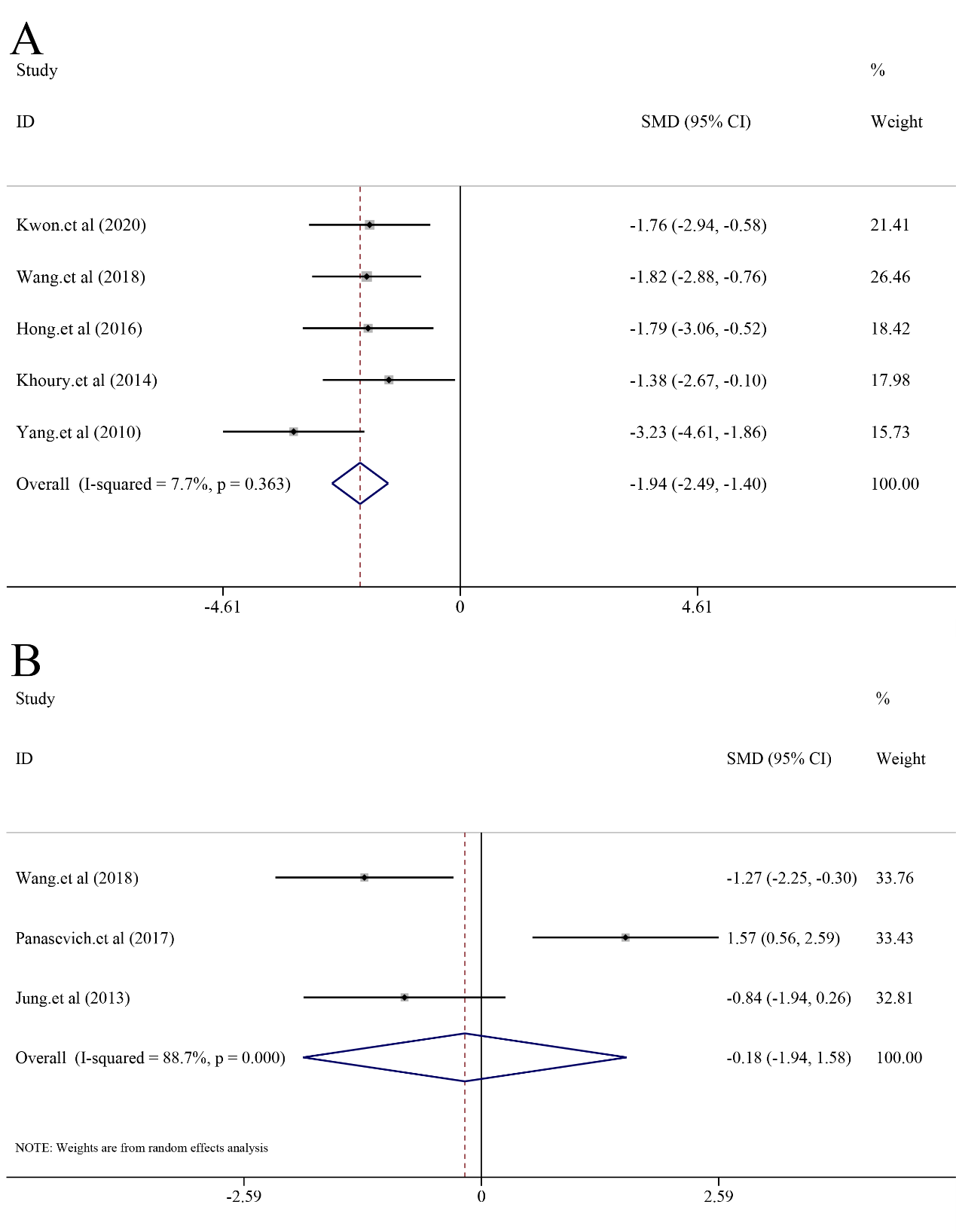


**Supplementary Figure 6. Effect of soybean on TNF-α and Insulin level in preclinical studies**

(A. Pooled effect of TNF-α; B. Pooled effect of Insulin)


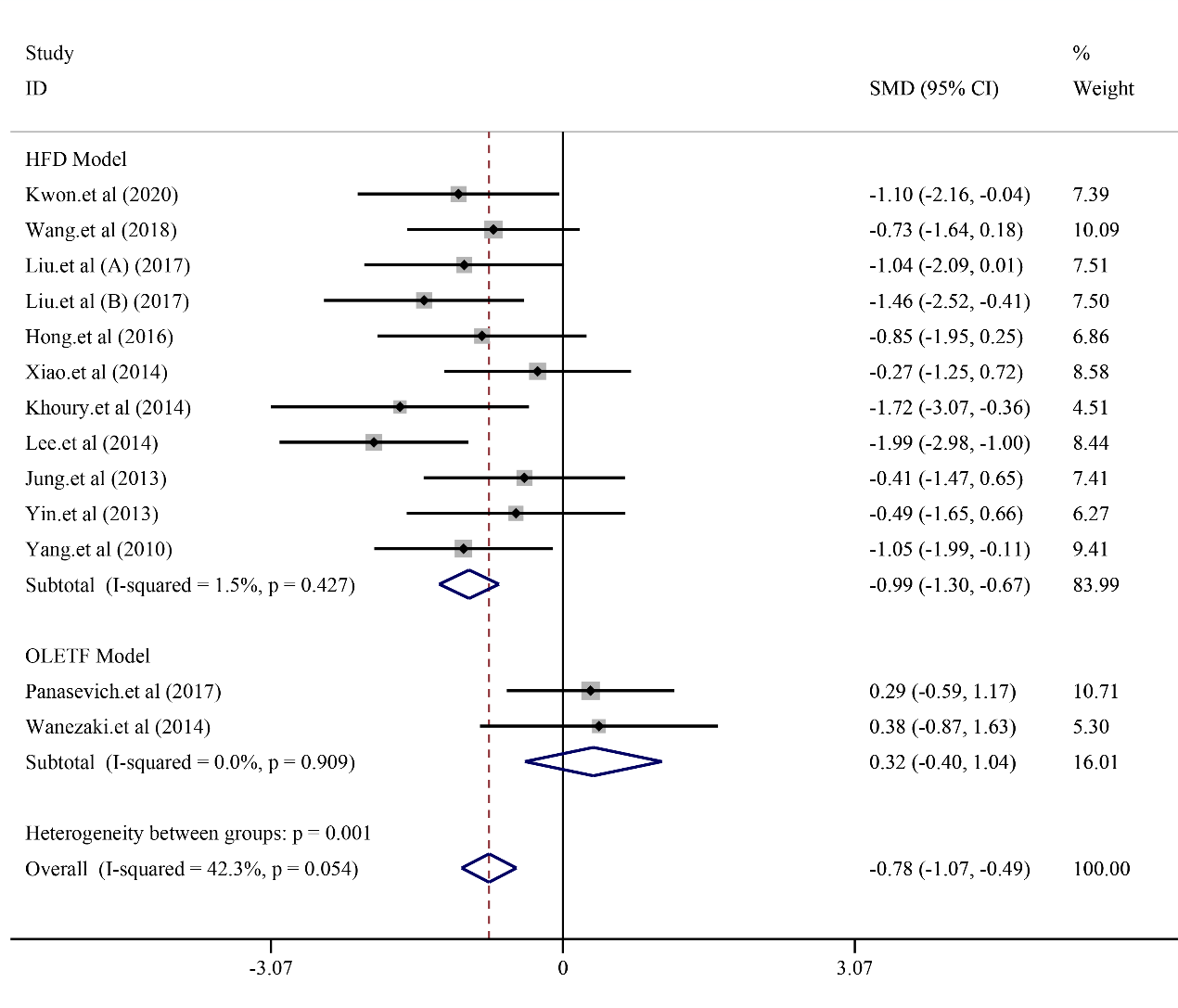


**Supplementary Figure 7. Subgroup analysis of TG based on animal model**


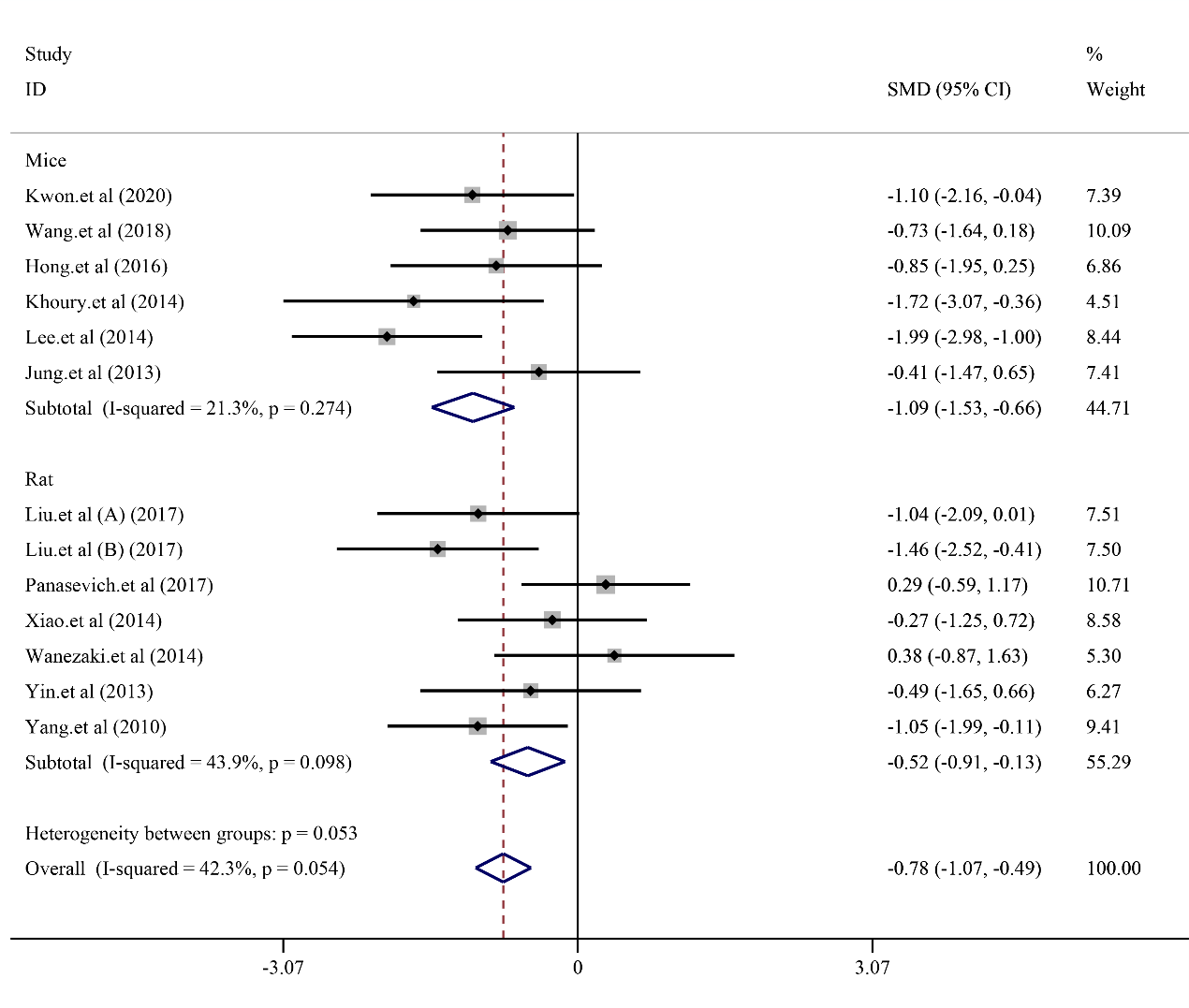


**Supplementary Figure 8. Subgroup analysis of TG based on animal species**


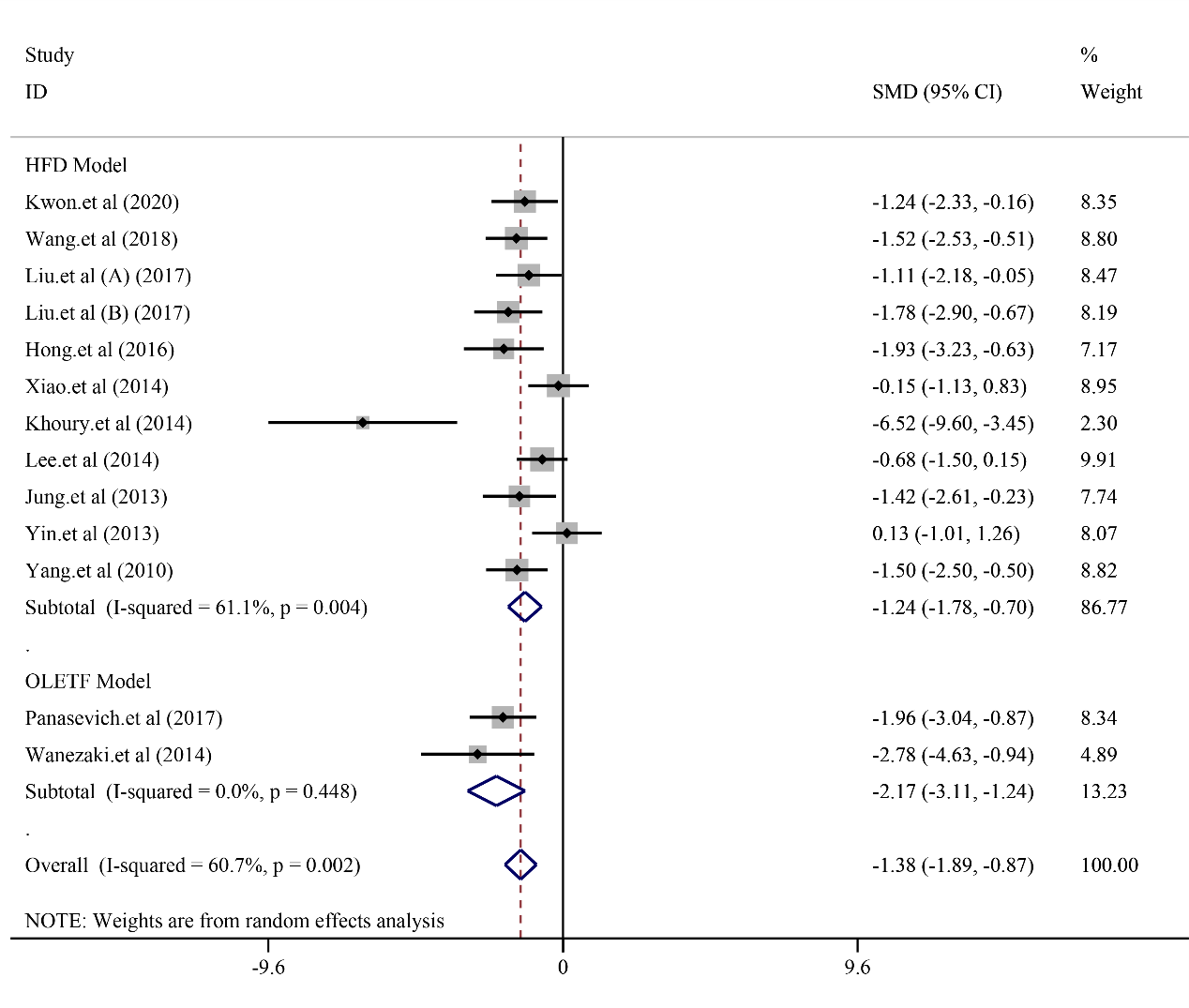


**Supplementary Figure 9. Subgroup analysis of TC based on animal model**


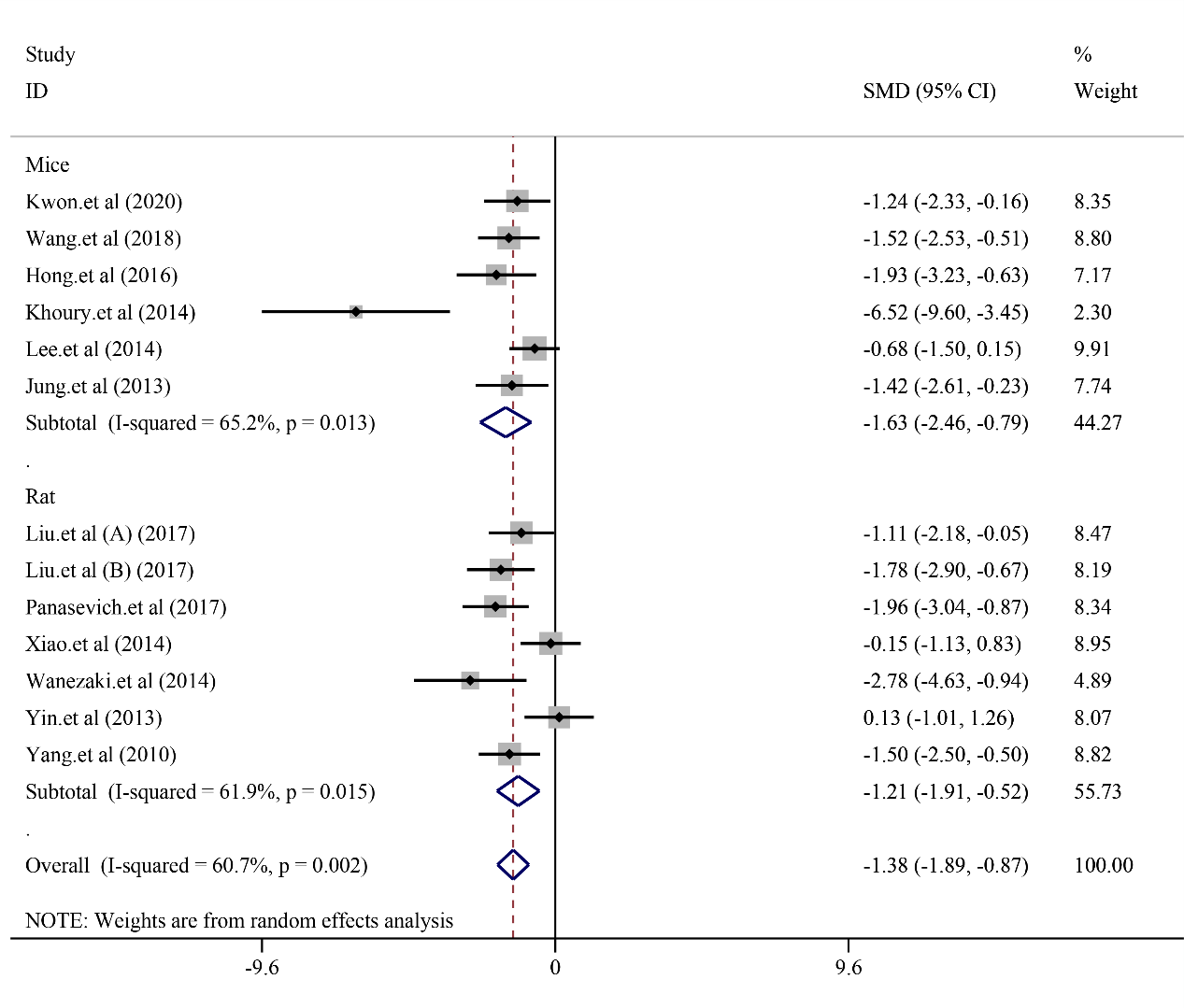


**Supplementary Figure 10. Subgroup analysis of TC based on animal species**


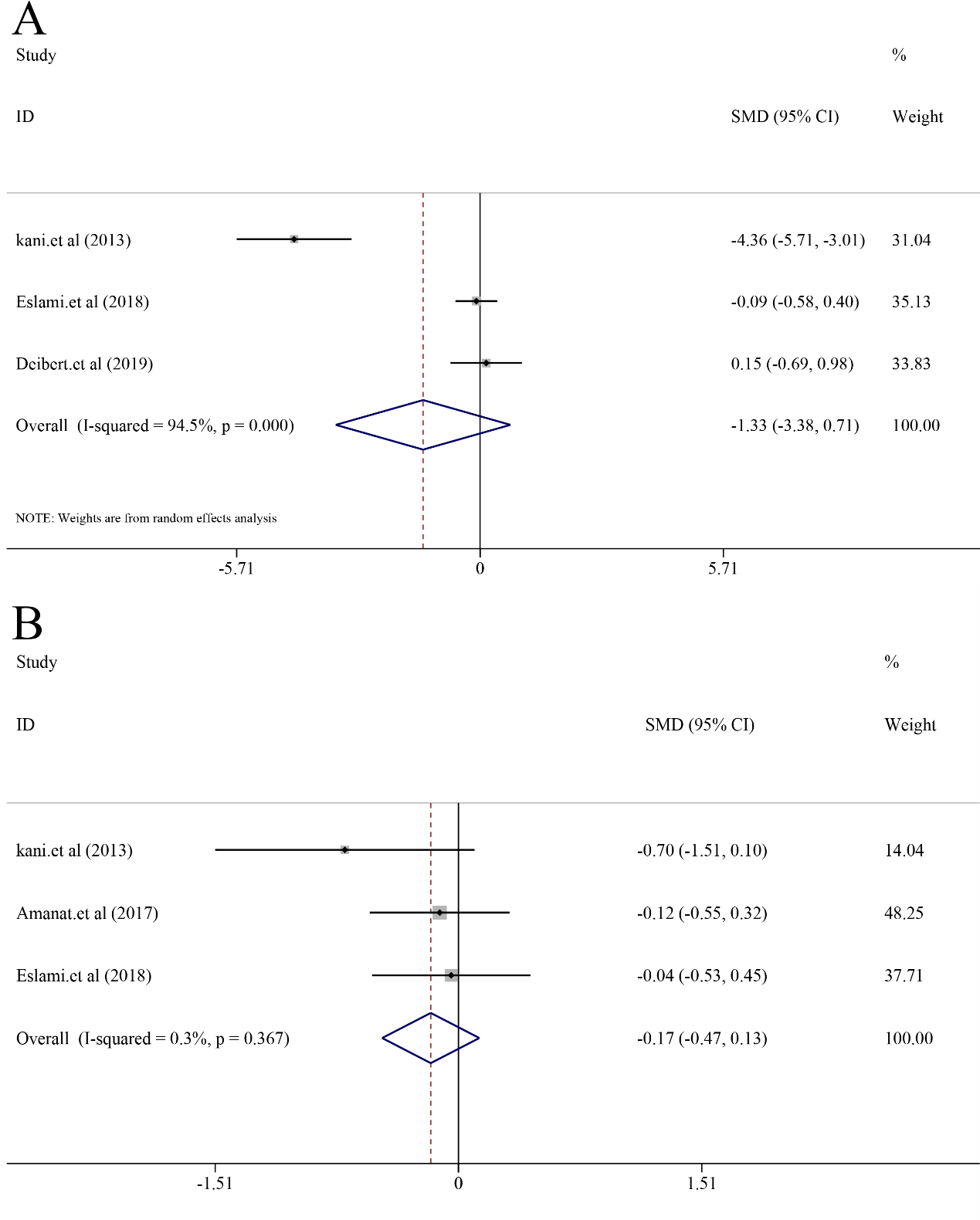


**Supplementary Figure 11 Effect of soybean on body weight and BMI in clinical trials**

(A. Pooled effect of body weight; B. Pooled effect of BMI)

**
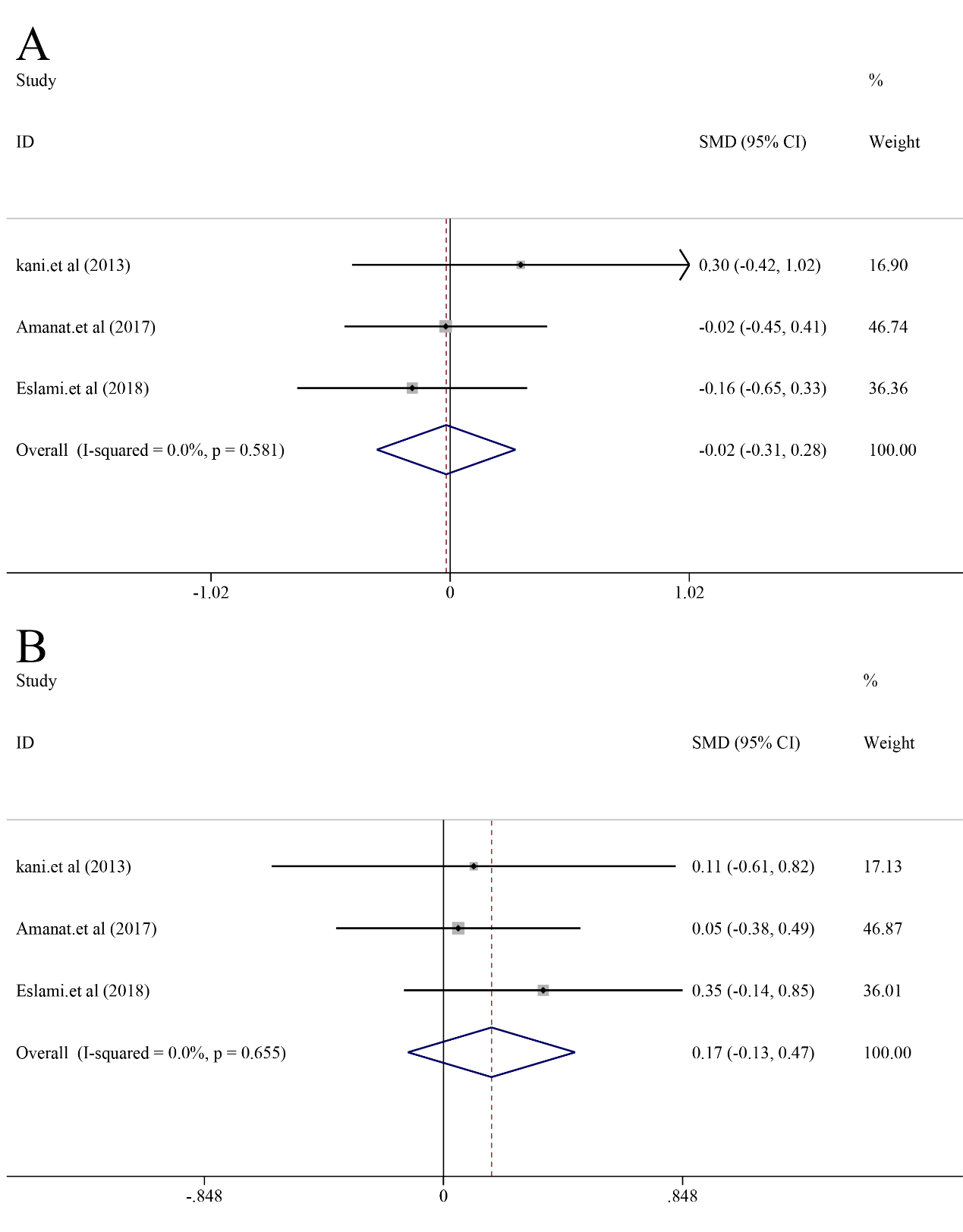
 Supplementary Figure 12. Effect of soybean on LDL-C and HDL-C level in clinical trials**

(A. Pooled effect of LDL-C; B. Pooled effect of HDL-C)


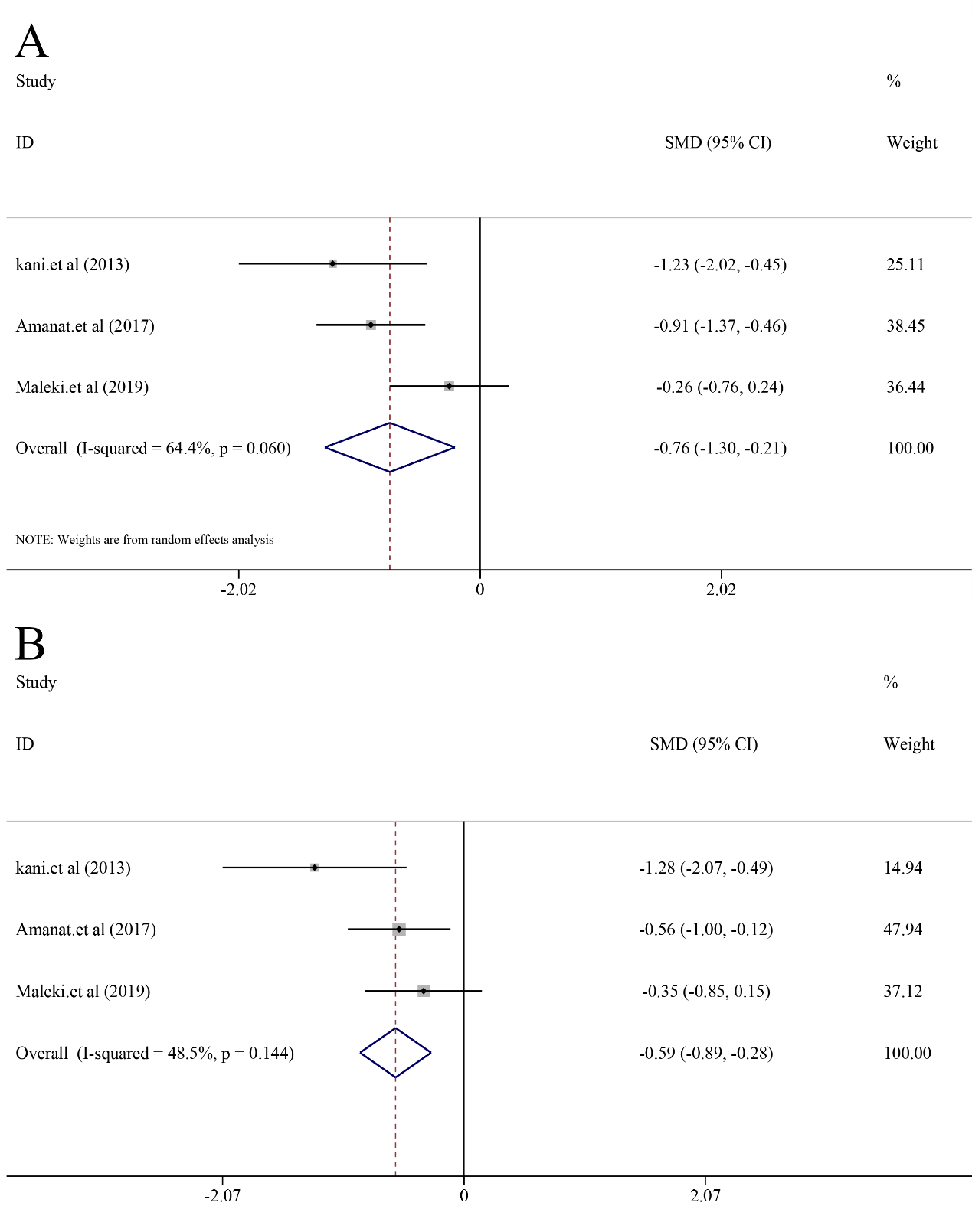
 **Supplementary Figure 13. Effect of soybean on MDA and Insulin level in clinical trials**

(A. Pooled effect of MDA; B. Pooled effect of Insulin)


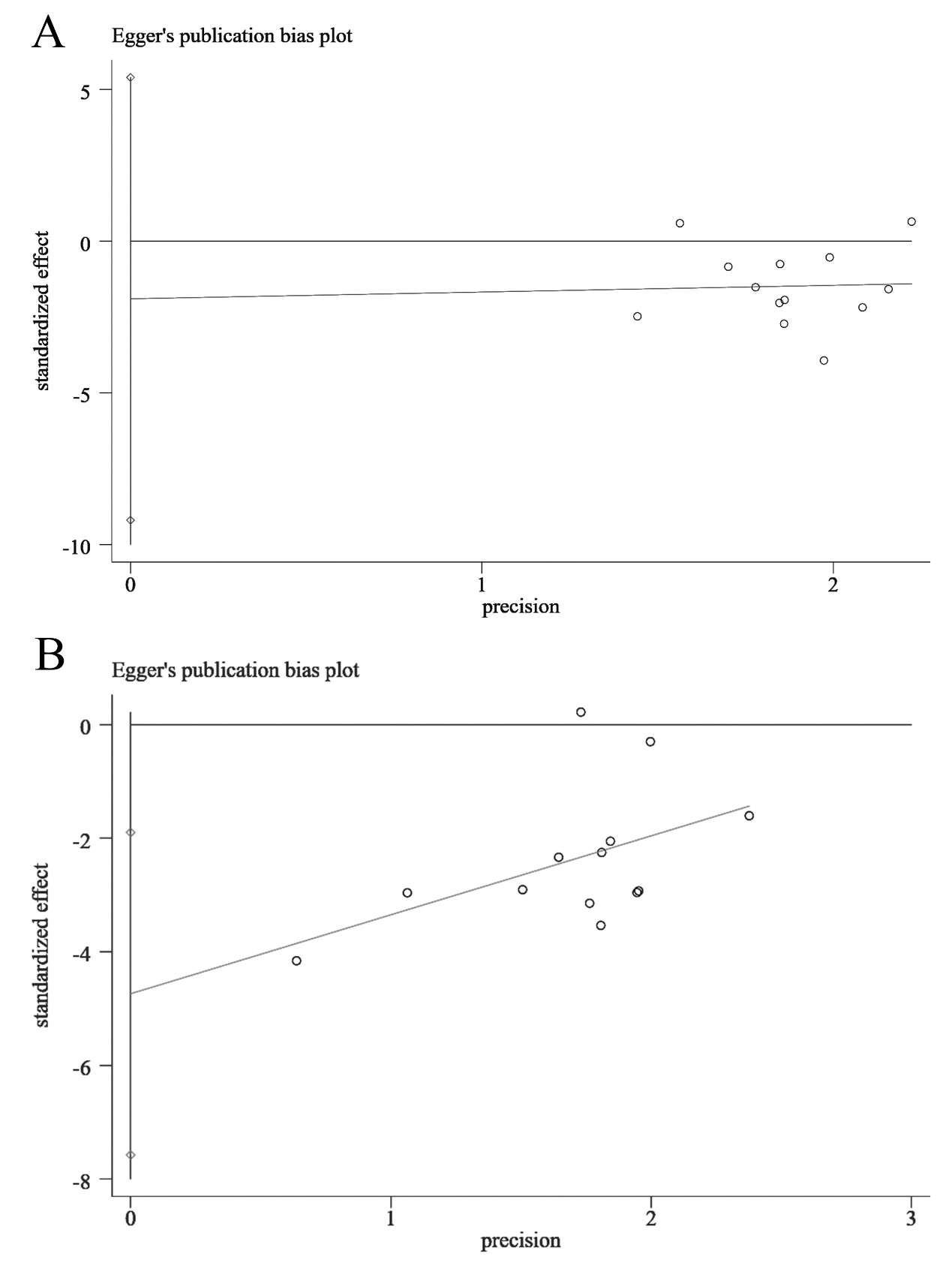


**Supplementary Figure 14. Egger’s test for TG and TC in preclinical studies**

(A. Egger’s test for TG; B. Egger’s test for TC)
